# Local Diversification of Methicillin- Resistant *Staphylococcus aureus* ST239 in South America After Its Rapid Worldwide Dissemination

**DOI:** 10.3389/fmicb.2019.00082

**Published:** 2019-02-27

**Authors:** Ana Maria Nunes Botelho, Maiana Oliveira Cerqueira e Costa, Ahmed M. Moustafa, Cristiana Ossaille Beltrame, Fabienne Antunes Ferreira, Marina Farrel Côrtes, Bruno Souza Scramignon Costa, Deborah Nascimento Santos Silva, Paula Terra Bandeira, Nicholas Costa Barroso Lima, Rangel Celso Souza, Luiz Gonzaga Paula de Almeida, Ana Tereza Ribeiro Vasconcelos, Apurva Narechania, Chanelle Ryan, Kelsey O’Brien, Sergios-Orestis Kolokotronis, Paul J. Planet, Marisa Fabiana Nicolás, Agnes Marie Sá Figueiredo

**Affiliations:** ^1^Laboratório de Biologia Molecular de Bactérias, Instituto de Microbiologia Paulo de Góes, Universidade Federal do Rio de Janeiro, Rio de Janeiro, Brazil; ^2^Laboratório Nacional de Computação Científica, Petrópolis, Rio de Janeiro, Brazil; ^3^Department of Pediatrics, Division of Pediatric Infectious Diseases, Children’s Hospital of Philadelphia and University of Pennsylvania, Philadelphia, PA, United States; ^4^Sackler Institute for Comparative Genomics, American Museum of Natural History, New York, NY, United States; ^5^Department of Epidemiology and Biostatistics, School of Public Health, SUNY Downstate Medical Center, Brooklyn, NY, United States

**Keywords:** methicillin-resistant *Staphylococcus aureus*, ST239-SCC*mec*III, Brazilian epidemic clone, Brazilian/Hungarian clone, comparative genomics

## Abstract

The global spread of specific clones of methicillin-resistant *Staphylococcus aureus* (MRSA) has become a major public health problem, and understanding the dynamics of geographical spread requires worldwide surveillance. Over the past 20 years, the ST239 lineage of MRSA has been recognized as an emerging clone across the globe, with detailed studies focusing on isolates from Europe and Asia. Less is known about this lineage in South America, and, particularly, Brazil where it was the predominant lineage of MRSA in the early 1990s to 2000s. To gain a better understanding about the introduction and spread of ST239 MRSA in Brazil we undertook a comparative phylogenomic analysis of ST239 genomes, adding seven completed, closed Brazilian genomes. Brazilian ST239 isolates grouped in a subtree with those from South American, and Western, romance-language-speaking, European countries, here designated the South American clade. After an initial worldwide radiation in the 1960s and 1970s, we estimate that ST239 began to spread in South America and Brazil in approximately 1988. This clone demonstrates specific genomic changes that are suggestive of local divergence and adaptational change including *agrC* single-nucleotide polymorphisms variants, and a distinct pattern of virulence-associated genes (mainly the presence of the *chp* and the absence of *sea* and *sasX*). A survey of a geographically and chronologically diverse set of 100 Brazilian ST239 isolates identified this virulence genotype as the predominant pattern in Brazil, and uncovered an unexpectedly high prevalence of *agr*-dysfunction (30%). ST239 isolates from Brazil also appear to have undergone transposon (IS*25*6) insertions in or near global regulatory genes (*agr* and *mgr*) that likely led to rapid reprogramming of bacterial traits. In general, the overall pattern observed in phylogenomic analyses of ST239 is of a rapid initial global radiation, with subsequent local spread and adaptation in multiple different geographic locations. Most ST239 isolates harbor the *ardA* gene, which we show here to have *in vivo* anti-restriction activity. We hypothesize that this gene may have improved the ability of this lineage to acquire multiple resistance genes and distinct virulence-associated genes in each local context. The allopatric divergence pattern of ST239 also may suggest strong selective pressures for specific traits in different geographical locations.

## Background

*Staphylococcus aureus* is a challenging human pathogen capable of causing a range of hospital (HA) and community-associated (CA) infections, varying from local and uncomplicated skin/soft tissue infections to more severe illnesses such as necrotizing fasciitis, pneumonia, bacteremia, osteomyelitis, and endocarditis. This extraordinary pathogenic potential is generally attributed to a plethora of well-known virulence-associated genes, and a relatively high rate of genomic plasticity that results in the frequent acquisition of new genes (Figueiredo and Ferreira, 2014). MRSA remains one of the leading causes of hospital infections worldwide, despite aggressive infection control measures (World Health Organisation [WHO], 2017). Interestingly, like CA-MRSA strains, the global diversity of HA-MRSA is represented by just a few, mostly clonal, epidemic lineages that appear to have adapted to specific challenges of the hospital environment (Hsu et al., 2015). In many cases, the specific factors that enhance the fitness of HA-MRSA strains are unknown, but it is likely that environmental and host challenges as well as inter-strain competition affect selection (Figueiredo and Ferreira, 2014). The mechanisms that lead to the spread of organisms over large geographical areas, even though they manifest mostly in hospitals, are also obscure, betraying a more complex epidemiology that includes large-scale waves of replacement of one predominant clone by another (Figueiredo and Ferreira, 2014; Hsu et al., 2015; Planet et al., 2017).

One of the most successful HA-MRSA lineages is the multilocus sequence type (ST) 239 that carries the staphylococcal cassette chromosome type III (SCC*mec*III). ST239-SCC*mec*III appears to have originated as a naturally occurring “hybrid,” from a large recombination event involving *S. aureus* ancestors of two major lineages, CC8 and CC30, which contribute about 80% and 20% of its genome, respectively (Robinson and Enright, 2004). The ST239-SCC*mec*III MRSA lineage has been associated with outbreaks in healthcare settings on all populated continents, and members of this lineage are generally characterized by their ability to cause serious, disseminated infections (Holden et al., 2010). Additionally, these bacteria present high-level resistance to many types of available antimicrobial drugs. Glycopeptide antibiotics and some more recent anti-MRSA-targeted antibiotics remain effective, but heteroresistance to vancomycin (hVISA) has recently been detected in ST239 isolates from Australia (van Hal et al., 2011).

Several studies have analyzed the genomic epidemiology and phylogeny of ST239 MRSA from different geographic regions (Harris et al., 2010; Yamamoto et al., 2012; Wang et al., 2014; Baines et al., 2015; Hsu et al., 2015; Arias et al., 2017). These studies focused mostly on isolates from Europe, Asia, and Australia; although a handful of ST239 strains from Latin America have been noted to form their own clade. Within this clade is a ST239 lineage referred to as the Brazilian epidemic clone (BEC) that was first reported in 1992 (Teixeira et al., 1995). For over 20 years this clone has been one of the most common MRSA isolates collected in Brazilian hospitals (Figueiredo and Ferreira, 2014). To gain a better understanding of this lineage we completed, closed, and annotated genomes for seven ST239 isolates of Brazilian MRSA (ST239-BR_C_). We used publicly available ST239 genome sequences to assess the origin and phylogeography of these isolates, and to generate a more complete picture of the spread and local adaptation of ST239.

## Materials and Methods

### Bacterial Strains and Genomes

Seven representatives of the ST239-SCC*mec*III lineage obtained from clinical samples in Brazil, including *agr*-functional and -dysfunctional isolates, were selected for whole-genome sequencing. These MRSA isolates were collected from patients presenting with local or invasive hospital-associated (HA) infections, admitted at different public general hospitals; and from colonization cases involving patients enrolled in a home-care system, from 1993 to 2001, as indicated in Table 1. The strains BMB9393, HC1335, and HC1340 were isolated in Rio de Janeiro, RJ (southeast region of Brazil), GV69, GV51, and GV88 in Teresina, PI (Northeast region), and Be62 in Belém, PA (North region). The distance from Rio de Janeiro to Belem is about 2,464 km and from Belém to Teresina approximately 752 km. The genomes of seven of these Brazilian ST239 MRSA were completely closed and referred collectively as **ST239-BR_C_**.

**Table 1:** Clinical source, public collection and GenBank accession numbers of the ST239 MRSA strains from Brazil sequenced.

	Isolation	Geographic		Agr	Public collection	GenBank accession
Strains	year	information	Source	function	accession number^∗^	number
BMB9393	1993	Rio de Janeiro	Blood	+	P4523	CP005288
GV69	1996	Teresina	Skin wound	-	P4521	CP009681
GV88	1997	Teresina	Skin wound	+	P4522	CP012018
Be62	1996	Belém	Blood	+	P4524	CP012013
GV51	1997	Teresina	Bronchial lavage	+	P4520	CP012015
HC1335	2001	Rio de Janeiro	Nasal swab	-	P4517	CP012012
HC1340	2001	Rio de Janeiro	Nasal swab	-	P4518	CP012011


*^∗^These strains are deposited at the CMRVS public collection at Fundação Oswaldo Cruz (www.portal.fiocruz.br).*

Additionally, a collection of 100 hospital MRSA isolates previously genotyped as ST239-SCC*mec*III (Teixeira et al., 1995) from different geographic regions of Brazil was used in experimental assays. We refer to this collection as **ST239-BR_100_**. These isolates were collected from sites of infection (*n* = 62) or from nasal colonization (*n* = 38), and only one isolate per patient was included. The main characteristics of these isolates are listed in Supplementary Table S1.

For detailed comparative genomic analyses, we used two other representative genomic subsets (**ST239-INT_C_**, **non-ST239-INT_C_**). First we defined an international subset of completed and annotated ST239 genomes from GenBank (**ST239-INT_C_**). These genomes were from the following isolates: (i) TW20 (GenBank Accession Number: FN433596), isolated in 2003 from a 2-year MRSA outbreak in an intensive care unit (ICU), in London (Holden et al., 2010); (ii) JKD6008 (Acc: CP002120), a vancomycin-intermediate *S. aureus* (VISA) strain obtained in 2003 from blood after 42 day-vancomycin treatment, in New Zealand (Howden et al., 2010); (iii) T0131 (Acc: CP002643), isolated in 2006 from an 87-year-old in China (Li et al., 2011); (iv) Z172 (Acc: CP006838), a VISA isolate obtained in 2010 from blood of an elderly patient in an ICU in Taiwan (Chen et al., 2013) and (v) XN108 (Acc: CP007447), also a VISA isolate collected from a burned patient with a wound infection, in China (Zhang et al., 2014). We also used another comparative group of well-described, international, complete HA-MRSA genomes from outside of the ST239 lineage (**non-ST239-INT_C_**), which included the following genomes: (i) MRSA252 (Acc: BX571856), a ST36-SCC*mec*II isolate, representative of the EMRSA-16 clone (Holden et al., 2004); (ii) Mu50 (Acc:: BA0000 17), a ST5-SCC*mec*II isolate, representative of the USA100 clone (Kuroda et al., 2001); (iii) CA-347 (Acc: CP006044), a ST45-SCC*mec*IV isolate, representative of the USA600 clone (Stegger et al., 2013); (iv) H-EMRSA-15 (Acc: CP007659), a ST22-SCC*mec*IV isolate, representative of the EMRSA-15 clone (Sabirova et al., 2014), and (v) 2395 (Acc: CP007499), a ST8-SCC*mec*IV isolate, representative of the USA500 clone (Benson et al., 2014). The characteristics of ST239-INT_C_ and non-ST239-INT_C_ are summarized in Table 2.

**Table 2:** General characteristics of the closed genomes of the HA–MRSA strains from ST239 and non-ST239 lineages.

		Chromosome		Number of genes	Plasmid
Isolate	Lineage	size (Mbp)	%GC	in chromosome	(Kbp)	tRNA	Reference
BMB9393	ST239-SCC*mec*III	2.98	32.9%	3,073	2.9	60	This study
GV69	ST239-SCC*mec*III	3.05	33.0%	3,175	–	60	This study
GV88	ST239-SCC*mec*III	2.98	32.9%	2,954	2.9	57	This study
Be62	ST239-SCC*mec*III	2.99	32.9%	2,951	2.7	61	This study
GV51	ST239-SCC*mec*III	2.98	32.9%	2,965	2.9	60	This study
HC1335	ST239-SCC*mec*III	2.97	32.9%	2,958	–	60	This study
HC1340	ST239-SCC*mec*III		32.9%	3,026	–	60	This study
TW20	ST239-SCC*mec*III	3.04	32.8	3,172	29.6/3	60	Holden et al., 2010
JKD6008	ST239-SCC*mec*III	2.92	34.0	3,026	–	82	Howden et al., 2010
T0131	ST239-SCC*mec*III	2.91	32.8	2,976	–	54	Li et al., 2011
Z172	ST239-SCC*mec*III	2.99	32.8	3,125	27.3/3	60	Chen et al., 2013
XN108	ST239-SCC*mec*III	3.05	32.8	3,049	–	57	Zhang et al., 2014
MRSA252	ST36-SCC*mec*II	2.90	32.8	2,939	–	59	Holden et al., 2004
Mu50	ST5-SCC*mec*II	2.88	32.9	2,958	25.1	59	Kuroda et al., 2001
2395	ST8-SCC*mec*IV	2.96	31.9	3,104	32.4	59	Benson et al., 2014
CA-347	ST45-SCC*mec*IV	2.85	32.9	2,696	24.7	60	Stegger et al., 2013
H-EMRSA-15	ST22-SCC*mec*IV	2.85	32.8	2,662	–	57	Sabirova et al., 2014


Besides the seven genomes sequenced for this work (**ST239-BR_C_**) and the five completely closed genomes available on NCBI (**ST239-INT_C_**), a total of 171 ST239 and 7 single-locus variant (SLV) raw genome sequences deposited in the GenBank^1^, along with those used in the phylogenetic studies published by Castillo-Ramírez et al. (2012) and Harris et al. (2010) deposited in the EMBL-Bank (Acc: ERA000102) were also used in this study for phylogenetic analyses, altogether totaling 190 genomes. Only genomes of strains with reported isolation date were considered for the molecular clock analysis (*n* = 167 genomes). The accession numbers for these assemblies are listed in Supplementary Table S2. For comparative genomic studies, the genome of the Brazilian strain BMB9393 was used as reference, in order to easily highlight the differences between Brazilian and international ST239 genomes. The strain TW20 that belongs to the ST239 lineage and has been used as reference in genomic studies of ST239 MRSA was chosen as the reference for phylogenetic analyses.

### Whole Genome Sequencing and Assembly

DNA from the seven **ST239-BR_C_** MRSA isolates used was obtained by phenol extraction and ethanol precipitation (Sambrook et al., 1989). The concentration and purity of the DNA were assessed using a Qubit^®^ 2.0 fluorometer (Invitrogen, Eugene, OR, United States). A total of 5 μg genomic DNA was used to prepare a paired-end library. The genome sequencing was performed using a 454 GS FLX Titanium (3-kb paired-end library) approach (Roche Diagnostics Corporation, Indianapolis, IN, United States). The assembly was accomplished using Newbler v2.6 (Roche) (Margulies et al., 2005) and Celera Assembler v6.1 (Myers et al., 2000). Gaps within scaffolds resulting from repetitive sequences were resolved by *in silico* gap filling. The complete genome assembly was accomplished using a combination of Newbler v 2.6 (Roche Inc.) and GapFiller (Boetzer and Pirovano, 2012). The consensus sequences assembled into contigs and scaffolds were aligned against a database of complete genomes from the *Staphylococcus* genus to determine the orientation and order of the contigs and scaffolds (Supplementary Figure S1). The gaps, intra- and inter-scaffolds, were filled by individual assemblies of the reads falling in both termini of a given gap (Supplementary Figure S1). This was accomplished by selecting reads that formed the end of contigs adjacent to each gap stretches. Those reads were assembled separately with Newbler. Contiguous sequences generated by this approach that were able to complete the gap and anchor on the two adjacent contigs were added to the sequence, thus closing the gap. As a last step of gap closure, the remaining gaps were closed using the GapFiller program.

### Genome Annotation

Genome annotation was performed using the System for Automated Bacterial Integrated Annotation (SABIA) (Almeida et al., 2004). This software uses an automated annotation pipeline, where each open-reading frame (ORF) is submitted for comparison with several databases (NCBI-nr, KEGG, InterPro, and UniProtKB/Swiss-Prot), and the results are made available on the screen for assessment by expert users. All possible ORFs were predicted by Glimmer (Delcher et al., 2007) and GeneMark (Besemer and Borodovsky, 2005) and the transfer RNAs (tRNAs) by tRNAscan-SE (Schattner et al., 2005). The identification of bona fide ORFs and their probable functions included similarity searches using both nucleotide and amino acid sequences by Basic Local Alignment Search Tool (BLAST) against KEGG, NCBI-nr, and UniProtKB/Swiss-Prot databases, as well as identification of protein domains and important sites using InterPro (Mitchell et al., 2015).

### Comparative Genomics Analysis

Chromosomal architecture and genome organization were initially analyzed using ProgressiveMauve (Darling et al., 2010), with default parameters. The inversions found in the GV69 genome (ST239-BR_C_), using BMB9393 genome (ST239-BR_C_) as reference, were confirmed by a PCR-based assay using specific primers listed in Supplementary Table S3. Thus, to confirm the position of each locally collinear block (LCB) in the GV69 genome, primers were designed using the genome of GV69 based on contiguous sequences of adjacent LCBs. The rearrangement found *in silico* was confirmed when the PCR resulted in the expected amplicon size. The MicroScope platform (Vallenet et al., 2009, 2013) was used for determination of the RGPs and unique/shared gene content identification. RGPs were defined as DNA segments over 5 kbp that were possibly related to events of horizontal exchanges. Additional features for identification of RGPs included G + C% deviation, compositional biases, presence of prophage genes, synteny breaks, and proximity to tRNAs. For these analyses, the BMB9393 chromosome was set as the reference and RGPs were mapped in ST239-BR_C_, ST239-INT_C_, and non-ST239-INT_C_ genomes. Each identified RGP was manually inspected based on the genomic context and conserved genes located at the flanking regions. Other genomic analyses were performed using the comparative genomic tools available in SABIA platform (Almeida et al., 2004). The Geneious software (Biomatters Ltd., Auckland, New Zealand) was also used for genome alignment and visualization of the regions analyzed. Putative bacteriophages were detected using the phage search tool, PHAST (Zhou et al., 2011). The set of virulence genes (virulome) and staphylococcal pathogenicity island (SaPI) and other genomic islands (GIs) were detected using VirulenceFinder 1.5 (minimum length = 80%, threshold id = 98%) and manual inspection using Uniprot/Swissprot, BLAST, and literature review (Joensen et al., 2014). The EasyFig software (Sullivan et al., 2011) was used for creating linear comparative figures of multiple genomic loci, and the BLASTn atlas was generated with GVIEW, applying as parameters identity >80% and *e*-values <10^-10^ (Petkau et al., 2010).

We used PCR to search for important genomic features in the whole collection of **ST239-BR_100_** isolates. The primers used for each assay are listed in Supplementary Table S3.

### Phylogenetic Analysis and Divergence Times

Besides the genomes sequenced in this study (**ST239-BR_C_**) and the international representative genomes used (**ST239-INT_C_**), ST239 genomes were downloaded from NCBI or from EMBL (*n* = 190) and were used for whole-genome alignment with reference to the *S. aureus* TW20 genome, using the NUCmer and show-snps utilities of MUMmer^2^. All regions from the reference genome annotated as MGEs were excluded as well as the approximately 20% of the genome thought to be derived from CC30 (as in Holden et al., 2010; Supplementary Table S4). We also applied a mask that excluded repetitive sequences from the reference genome that were >80% identical over at least 100 nucleotides to other genomic loci, based on pairwise MegaBLAST-based analysis. Phylogenetic reconstruction was carried out by using the alignment of polymorphic sites in IQ-TREE v1.6.2 (Nguyen et al., 2015). One BIONJ (Gascuel, 1997) and 100 parsimony trees were used as starting trees for the maximum-likelihood (ML) tree search. The substitution model implemented was the general time-reversible (GTR) (Lanave et al., 1984) and empirical base frequencies, among-site rate heterogeneity was modeled according to the free-rate model (Yang, 1995; Soubrier et al., 2012), and ascertainment bias for variable-only sites was corrected (Lewis, 2001). Internode branch support was evaluated with the ultrafast bootstrap approximation method (UFBoot) and a maximum of 1000 replicates (Minh et al., 2013), using a convergence criterion, as well as with the Shimodaira–Hasegawa–aLRT branch test (Guindon et al., 2010) and 1,000 replicates.

To estimate the time that elapsed since the genomic divergence of the isolates in our dataset, we employed a Bayesian phylogenetic framework implemented in BEAST v2.5.1 (Drummond et al., 2012). For this analysis, genomes without collection date report were excluded, leading to a total of 167 genomes included. In addition to the above masked regions that were potentially mobile (Supplementary Table S4), we sought to exclude any regions with evidence of recombination. For this analysis, we rebuilt the genome matrix using simulated paired-end reads that were generated for the 167 assembled genomes that have dates using wgsim^3^ in the haplotype mode without introducing errors, mutations, or indels. Read mapping to TW20 reference genome and variants calling were performed using Snippy v.4.3.3^4^. The regions in TW20 excluded in the previous step were masked in this analysis as well. A whole genome alignment, including SNPs and invariant sites, produced by Snippy was used to infer an initial phylogenetic tree in RAxML v8.2.4 (Stamatakis, 2014) using the GTR substitution model (Lanave et al., 1984) accounting for among-site rate heterogeneity using the Γ distribution and four rate categories (Yang, 1995) for 100 individual searches with maximum parsimony random-addition starting trees. Node support was evaluated with 100 nonparametric bootstrap pseudoreplicates (Felsenstein, 1985). The initial ML newick tree and the whole-genome alignment were used as input for ClonalFrameML (Didelot and Wilson, 2015) to infer recombination using 100 pseudo-bootstrap replicates. The maskrc-svg script^5^ was then used to mask recombinant regions from the original whole-genome alignment. A SNP alignment was then called from the resultant alignment, from maskrc-svg, using the SNP-sites tool (Page et al., 2016).

The SNP alignment was then used to estimate divergence times in BEAST v2.5.1 (Bouckaert et al., 2014). In order to gauge the potential for temporal signal of our dataset, we used a regression of root-to-tip genetic distance vs. isolation time as a diagnostic tool, as implemented in TempEst v1.5.1 (Rambaut et al., 2016). A positive correlation between genetic divergence and isolation time was observed (plot not shown; *R*^2^ = 0.6413), indicating suitability of this dataset for downstream molecular clock analysis in BEAST. The Hasegawa–Kishino–Yano nucleotide substitution model was used (Hasegawa et al., 1985) with among-site rate heterogeneity modeled with the Γ distribution and four discrete rate categories (Yang, 1995), and ascertainment bias for variable-only sites was corrected by factoring in the number of invariable sites based on fully sequenced MRSA genomes. We implemented an uncorrelated lognormal relaxed clock model with a random starting tree, a Bayesian skyline coalescent (Drummond, 2005), and a uniform prior probability distribution of 10^-4^–10^-8^ substitutions/site/year. The chain length was set at 200 million MCMC steps with a 1,000-step thinning. The sampled parameters and their effective sample size (ESS > 200) were inspected in Tracer v1.6^6^. LogCombiner was then used to resample posterior distribution of the trees file at a lower frequency (10,000 steps). We also implemented a strict clock in BEAST with a random starting tree, a Bayesian skyline coalescent (Drummond, 2005), and a uniform prior probability distribution of 10^-4^-10^-8^ substitutions/site/year. The chain length was set at 50 million MCMC steps with a 5,000-step thinning. Two independent runs were combined post convergence, following inspections of the sampled parameters and their ESS. The chronograms were plotted on the basis of the maximum clade credibility tree using the TreeAnnotator program from the BEAST package and were visualized in FigTree v1.4.3^7^. The trees and divergence times produced by the strict and relaxed clock techniques were largely indistinguishable.

### Detection of Agr Activity in the ST239 Isolates

The detection of Agr activity in the collection of one hundred (ST239-BR_100_) isolates was performed using MALDI–TOF–MS according to a previously described method (Josten et al., 2014). Briefly, individual colonies were subjected to MALDI-TOF-MS for detection of a peak at *m*/*z* 2415 representing the phenol soluble modulin PSM-*mec*, an Agr-regulated gene harbored on some types of SCC*mec*, including SCC*mec*III, which is carried by the ST239 lineage.

Strains BMB9393 and HC1335 were also tested using a real-time quantitative reverse transcription-polymerase chain reaction (real-time qRT-PCR) for the RNAIII transcript with expression of the 16S rRNA gene as a control. For RNA preparations, bacterial cells grown in BHI broth (18 h; 37°C; 250 rpm) were collected in the stationary phase. Total RNA was prepared using RNeasy Mini kit (Qiagen) and quantified using a Nanodrop Lite Spectrophotometer (Thermo Scientific, ThermoFisher Scientific). RNA integrity was analyzed by gel electrophoresis. Real-time qRT-PCR was performed using the Power SYBR Green RNA-to-CT^TM^ 1-Step Kit (Applied Biosystems, ThermoFisher Scientific) as recommended. The run was performed in a StepOne Real-Time PCR System (Applied Biosystems, ThermoFisher Scientific) and analyzed using the StepOne Software v2.2 (Applied Biosystems, ThermoFisher Scientific). The cycling conditions for all primers were performed as follows: 48°C for 30 min (cDNA preparation); 95°C for 10 min; 35 cycles of 95°C for 30 s, 55°C for 45 s, and 72°C for 45 s (cDNA amplification cycles). At least three biological replicates were run with four technical replicates each. Primers used are listed in Supplementary Table S3.

### IS*256* Insertion in *mgrA* Promoter Region and in the *agrC* ORF

Genomic analysis showed an IS*256*-related element upstream *mgrA* ORF in the genome of BMB9393 strain (ST230-BR_C_). The frequency of this insertion in our collection of 100 ST239-BR isolates was assayed using primers designed based on the BMB9393 genome sequence, with the forward primer in the IS*256* sequence and the reverse in the *mgrA* sequence. Likewise, the IS*256* insertion found disrupting *agrC* ORF in the genome of HC1335 (ST239-BR_C_) was also searched for in the whole ST239-BR collection using a PCR-based approach, with forward primer in the *agrC* sequence and the reverse in the IS*256* sequence (based on the HC1335 genome sequence). Amplicons with the expected sizes were considered positive for the correspondent insertion.

### Detection of Recombination and Natural Selection in *agrC*

We mined *agrC* coding sequences from all assembled genomes and were condensed into 15 unique haplotypes. Those were then aligned by coding frame using MAFFT v7.407 (Katoh and Standley, 2013), and first checked for recombination breakpoints using the GARD algorithm (Kosakovsky Pond et al., 2006) on the DataMonkey web server^8^. We then employed several tests for positive or diversifying selection at different levels on this dataset. First, we examined whether or not over the length of the gene and across the phylogeny there had been at least one codon site that experienced diversifying selection on a branch. This was done using Branch-Site Unrestricted Statistical Test for Episodic Diversification (BUSTED) as a gene-wide test by testing all branches across the tree (Murrell et al., 2015). We then used different methods to investigate the potential for diversifying selection having acted on individual codon sites and, separately, on branches of the phylogeny. We employed two sitewise methods: Mixed Effects Model of Evolution (MEME) (Murrell et al., 2012) and Fast Unconstrained Bayesian AppRoximation (FUBAR) (Murrell et al., 2013), asking whether the intensity of natural selection has been relaxed or increased along internal branches of the phylogeny using the RELAX framework (Wertheim et al., 2015).

### ArdA Expression in the *ardA*-Negative *S. aureus* Strain RN4220

The *ardA* sequence was amplified using the primers *ardA*-fwd and *ardA*-rev (Supplementary Table S3) and genomic DNA from *S. aureus* BMB9393 as template. The *ardA* gene was initially cloned into the p-GEM T easy vector (Promega) and subcloned into the expression vector pCN40 using *Bam*HI and *Eco*RI restriction sites (Charpentier et al., 2004). The recombinant plasmid pCN40A (pCN40:P*_blaZ_-ardA*) or empty pCN40 were transformed by electroporation into DC10B competent cells (Monk et al., 2012). The recombinant plasmids (pCN40A) and pCN40 were obtained (QIAfilter Plasmid Midi Kit; Qiagen), and transformed into RN4220 by electroporation (Monk et al., 2012), yielding the clones 42P40E (RN4220; empty pCN40) and 42P40A (RN4220; pCN40A). Transformants were confirmed by DNA sequencing and the expression of *ardA* in 42P40E was detected using real-time qRT-qPCR.

### Competence Assays

The natural *S. aureus* plasmid pBMB (Costa et al., 2013) and the shuttle-vector pLI50 (Addgen) were obtained from *S. aureus* BMB9393 and *Escherichia coli* DC10B strains, respectively. 42P40E or 42P40A competent cells were submitted to electroporation with 0.1 mg/μl of pBMB and 10 mg/μl of pLI50. Chloramphenicol-resistant colonies derived from 42P40A or 42P40E were measured as colony forming units (CFU)/ml.

### Statistical Analysis

We used Student’s *t*-test (α = 0.05) where noted. In addition to the selection tests noted above, a Poisson distribution was used to determine the probability of a nucleotide change in *agrC* occurring at the same rate observed for other ST239 *S. aureus* virulence regulator genes. To determine the most representative genetic pattern of a clade, defined by presence/absence of the loci (*sasX*, *sae*, *chp*, and *agrC* clade-specific SNPs), we used the Grubbs’ test. Subsequently, a χ^2^-test for independence was used to test whether the main genetic pattern of a clade and its SLVs were associated with a specific clade. We also used a χ^2^-test to correlate these genetic patterns and broad geographic location (Eastern Mediterranean, Asia, South America). Because there are many sequenced genomes from Tunisia and Turkey, we randomly selected only three representative genomes of each of these two countries.

## Results and Discussion

### Genomic Characteristics and Chromosomal Architecture of ST239-BR_C_

**FIGURE 1:**
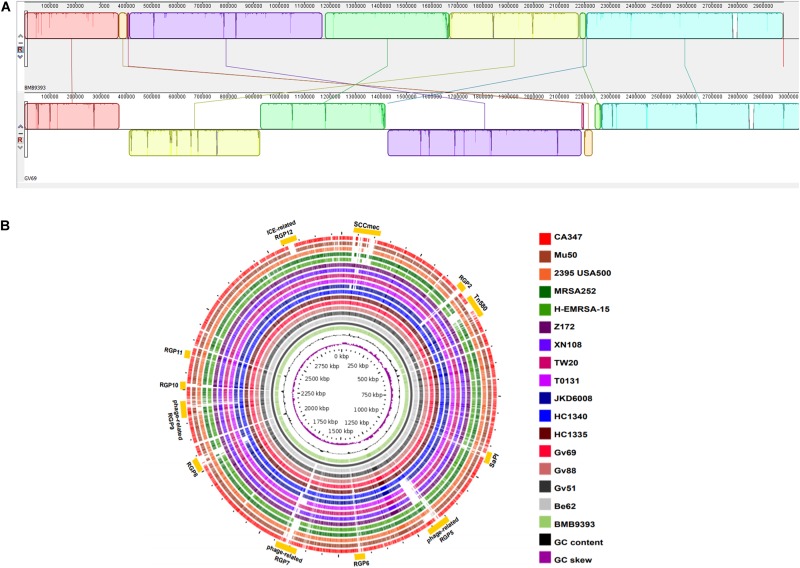
Genomic organization. **(A)** Genome alignments of ST239-BR_C_ strains BMB9393 and GV69 highlighting the distinct syntenic patterns among them. The genome of the strain BMB9393 (ST239-BR_C_) was used as reference. **(B)** The innermost ring (purple) plots the G+C skew of the reference, followed by its G+C content (black). The third ring represents the reference chromosome of strain BMB9393 and its coordinates. The following colored rings depict BLASTN (>80% identity and *e*-values < 10^-10^) comparisons obtained by GVIEW, between the chromosome of BMB9393 and those of ST239-BR_C_ strains (Be62, Gv51, Gv88, Gv69, HC1335, HC1340), ST239-INT_C_ (JKD6008, T0131, TW20, XN108, Z172), and non-ST239-INT_C_ (H-EMRSA-15, MRSA252, 2395, Mu50, CA-347). The outermost, interspaced rings (in dark yellow) represent the localization of the predicted RGPs in the BMB9393 chromosome, and the labels of each region as listed in Table 3.

To understand genomic diversity of our sequenced genomes (**ST239-BR_C_**), we did a detailed comparison to an international set of five completely closed ST239 genomes from different continents available in GenBank (**ST239-INT_C_**). We also defined a set of five other non-ST239, closed, and completed, *S. aureus* genome representatives from internationally disseminated lineages (**non-ST239-INT_C_**) commonly associated with hospital infections, including ST5, ST8, ST22, ST36, and ST45. The results of our analysis are shown in Figure 1.

The main characteristics of the completely sequenced and closed genomes of seven MRSA isolates from Brazil (**ST239-BR_C_**) are described in Table 2. Whole-genome alignments revealed a large genomic inversion in the GV69 genome compared to other **ST239-BR_C_** genomes (Figure 1A). This inversion was confirmed by PCR amplification of the expected products (see the section “Materials and Methods”). Additional chromosomal rearrangements were detected in comparison with **ST239-INT_C_** genomes (Figure 1B), as represented by breaks in the synteny between 10 genomic LCBs (Figure 1B).

### Unique and Shared Chromosomal Genes

Comparison of **ST239-BR_C_**, **ST239-INT_C_**, and **non-ST239-INT_C_** genomes performed using the SABIA platform (Almeida et al., 2004) revealed 1,991 genes in the ST239-SCC*mec*III core genome, defined here as the genes common to all ST239 completely closed genomes. When compared with the **non-ST239-INTc** genomes, only 11 ORFs were exclusively found among the **ST239-BR_C_** genomes studied. These unique ORFs are predicted to encode five distinct hypothetical proteins (BMB9393 SABB_01759, BMB9393 SABB_01754, BMB9393 SABB_01753, BMB9393 SABB_02921, and BMB9393 SABB_01926), a putative transposase (BMB9393 SABB_00433), the cassette chromosome recombinase A (BMB9393 SABB_01756), the ribosome-binding factor A (BMB9393 SABB_00236), an ATP-binding protein (BMB9393 SABB_02230), a putative phi PVL-like protein (BMB9393 SABB_04160), and a superantigen-like protein (BMB9393 SABB_02209).

A total of 82 ORFs were exclusively present in the **ST239-BR_C_** set compared with the **ST239-INT_C_** genomes (Supplementary Table S5): 19 (23.2%) encode hypothetical proteins, 40 (48.8%) encode putative proteins related to bacteriophages and other MGEs) and the remaining 23 (28%) ORFs have diverse predicted biological functions including some putative and known virulence-related genes, such as the gene for the chemotaxis inhibitory protein (*chp*; BMB9393 SABB_02361). A total of 59 ORFs (Supplementary Table S5) were exclusively found in ST239-INT_C_ genomes: 33 (55.9%) encode hypothetical proteins, 8 (13.6%) encode predicted proteins related to bacteriophages and other MGEs, and 18 (30.5%) encode putative proteins with different biological functions including the genes for known virulence-factors, such as enterotoxin A (Sea; TW20 SATW20_19410) and staphylococcal enterotoxin-like Q (SelQ; TW20 SATW20_08910). Most of these exclusive, or diagnostic, genes were found within RGPs, which will be discussed in the sections below.

**FIGURE 2:**
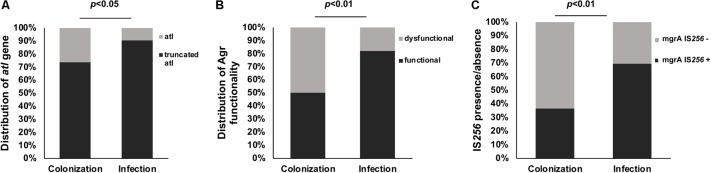
Differences in isolates collected from colonization and infection sites. **(A)** Percentage of truncated (black) and not truncated (gray) *atl* gene (PCR detected) in hundred ST239 isolates from Brazil collected from colonization and infection cases. **(B)** Distribution of functional (black) and dysfunctional (gray) Agr among 100 ST239 isolates obtained from colonization (*n* = 38) and infection cases (*n* = 62) in Brazil. **(C)** Presence (black) and absence (gray) of IS*256* insertion in the *mgrA* gene in 100 ST239 MRSA isolates from Brazil collected from colonization and infection cases. Note a significantly increased number of IS*256* insertion among infection cases compared with colonization cases (*p*-values calculated by Fisher’s exact).

The *S. aureus* autolysin gene *atl* (BMB9393 SABB_01019), in all **ST239-BR_C_** genomes, has a deletion of 243 bp in the coding region (Supplementary Figure S2A). PCR-based screening detected this deletion in 84% of the collection **ST239-BR_100_** clinical isolates. The truncated *atl* gene was significantly more prevalent in the group of **ST239-BR_100_** isolates that was collected from infection cases compared with that of colonization cases (*p* < 0.05), although this difference was relatively small (Figure 2A). It is possible that the absence of functional *atl* might be beneficial for immune evasion by reducing the amount of immune stimulation that is due to bacterial cell lysis (Humann and Lenz, 2009). It is possible that this could contribute to higher levels of invasive disease associated with these isolates.

### Regions of Genomic Plasticity (RGPs)

We identified 12 RGPs in Brazilian isolates through our comparative approach (Figure 1B). These RGPs contain multiple signatures of genomic plasticity and MGEs including sequences related to transposable elements, phage-structures, insertion sites for integrases or recombinases, as well as adjacent tRNA for insertions sites of GIs, as summarized in Table 3. The amount of DNA located in the RGPs accounted for at least 429.5 kbp or 14.4% of the chromosome of the BMB9393 reference genome. Another feature of these RGPs is that they have GC content typically equivalent to the chromosomal DNA, suggesting that HGT events have predominantly involved *S. aureus* or other bacteria with low GC content (Lindsay, 2008). The main characteristics of the most prominent RGPs are discussed below, with additional descriptions provided in Supplementary Table S6.

**Table 3:** RGPs identified comparing ST239 and non-ST239-closed genomes using the chromosome of the Brazilian strain BMB9393 as reference.

			Length			Best hit (% query coverage, % subject coverage,
Regions	Coordinates		(kbp)	GC%	Features	% identity, *e*-value)^∗^
RGP1	34139	105446	73.0	33.1	S.b., dGC%, Tnp, Rec, tRNA, GI SCC*mec*	*S. aureus* DNA, SCCmercury, SCC*mec* type III.1.1 (IIIA), complete sequence, strain OC3 (GenBank AB983237.1) (55%, 64%, 99%, 0.0)
RGP2	398966	413781	14.8	35.3	S.b., dGC%, Tnp	*S. aureus* NCTC13435 chromosome 1 (GenBank: LN831036.1) (100%, 0.6%, 99%, 0.0)
RGP3	451997	509822	57.8	33.3	S.b., dGC%, Int., Tnp, ICE, GI νSAβ	*S. aureus* subsp. *aureus* T0131, complete genome (GenBank: CP002643.1) (100%, 0.95%, 99%, 0.0).
RGP4	925027	939643	14.6	29.9	Ss.b., dGC%, Int, SaPI	*S. aureus* pathogenicity island SaPI Tokyo11212 (GenBank: AB860416.1) (84%, 72%, 98%, 0.0).
RGP5	1165376	1213829	48.5	33.6	S.b., dGC%, Rec, phage similar to YMC09	*Staphylococcus* phage phiSauS-IPLA35, complete genome (GenBank: EU861005.1) (61%, 65%, 97%, 0.0).
RGP6	1424422	1438004	13.5	28.1	S.b., dGC%, Tnp	*S. aureus* subsp. *aureus* Z172, complete genome (GenBank: CP006838.1) (100%, 0.2%, 100%, 0.0).
RGP7	1632358	1684127	51.8	34.6	S.b., dGC%, Int., phage similar to phiMR11	*Staphylococcus* phage 55, complete genome (GenBank: AY954963.1) (61%, 59% 98%, 0.0).
RGP8	2013514	2044432	30.9	28.9	S.b., dGC%, Tnp, tRNA, GI □SA□	*S. aureus* XN108, complete genome (GenBank: CP007447.1) (100%, 1.0%, 99%, 0.0).
RGP9	2149470	2222679	73.2	31.7	S.b., dGC%, Rec., phage similar to phiNM3, Tnp	*S. aureus* subsp. *aureus* ST72 strain TMUS2134, complete genome (59%, 1.4%, 99%, 0.0).
RGP10	2263898	2269345	5.4	31.1	S.b., dGC%, Tnp, tRNA	*S. aureus* BK16691, transposon Tn*4001* (GenBank: GU235985.1) (83%, 12%, 99%, 0.0).
RGP11	2353104	2378952	25.8	32.9	S.b., Tnp, Res, tRNA	*S. aureus* subsp. *aureus* TW20, complete genome (GenBank: FN433596.1) (100%, 0.8%, 99%, 0.0).
RGP12	2811924	2832078	20.2	29.6	S.b., dGC%, Tnp, ICE	*S. aureus* HDG2, integrating conjugative element ICE6013 (GenBank: FJ231270.1) (100%, 98%, 99, 0.0).


*^∗^BLASTN performed against the “nr” database at the NCBI website; ICE, integrative and conjugative element; SaPI, *S. aureus* Pathogenicity Island; Int., integrase; Rec., recombinase; Tnp, tranposase; Res, resolvase; dGC%, deviation from mean chromosomal GC content; s.b., synteny break from at least one other compared *S. aureus*; phage, probable bacteriophage region; tRNA, presence of flanking transfer RNAs; transposases, presence of transposase-related sequences. The complete sequence of each RGP identified in the reference genome BMB9393 was used as a query in the BLASTN against the NCBI. Query coverage corresponds to the sequence of each RGP in reference BMB9393. Subject coverage is the NCBI best hit sequence listed in the last column.*

#### RGP1

This region in **ST239-BR_C_** genomes carries two SCC elements, the SCC*mercury* (also known as SCCH*g*) and SCC*mec.* All ST239 closed genomes (ST239_C_) shared a SCC*mec* type III, although **ST239-BR_C_** and **ST239-INT_C_** display different architectures for this element. The **ST239-BR_C_** genomes carry SCC*mec*III.1.1.2 (named SCC*mec*IIIA), an approximately 28.0 kbp cassette, with 15-bp *att* direct-repeat sequences (*attL*, *attR*); however, this cassette is markedly distinct from TW20 (a representative of ST239 from the United Kingdom), which has SCC*mec*III.1.1.1 (approx. 35.3 kbp), as shown in Supplementary Figure S2B. The SCC*mec*III.1.1.1 (named SCC*mec*III) carries four copies of the IS*431* with the same orientation of those found in SCC*mec*III.1.1.2, suggesting that the SCC*mec*III.1.1.2 originated from a recombination event occurring between two IS*431* copies (Ito et al., 2001; Supplementary Figure S2B).

#### RGP3

In the 5′-portion of this RGP (Supplementary Figure S2C), there is an integrative and conjugative element (ICE) with similarity to Tn*5801*, while another segment corresponds to a GI of approximately 27.6 kbp known as νSAα. This GI, which is conserved among all ST239 genomes studied, carries a gene encoding a truncated transposase (BMB9393 SABB_02229) that shows high similarity to that of the IS*256* family; *hsdM* (BMB9393 SABB_05215) and *hsd*S (BMB9393 SABB_05408) genes encoding a restriction/modification system; a cluster of 10 *ssl* (staphylococcal superantigen-like) ORFs (BMB9393 SABB_02212 to SABB_02203) and putative ORFs encoding several lipoproteins (*lpl*) (BMB9393 SABB_02197 to SABB_05337). These two clusters are considered markers of the νSAα island (Baba et al., 2008).

In addition to *tetM*, this RGP also contains genes associated with conjugation, regulation, excision/integration systems, and a gene encoding the anti-restriction protein ArdA (BMB9393 SABB_02743). RGP3 showed sequence divergence for the genomes of the **non-ST239-INT_C_** (strains CA-347, H-EMRSA-15, and MRSA252) but it was highly conserved among all our comparison ST239 genomes.

**FIGURE 3:**
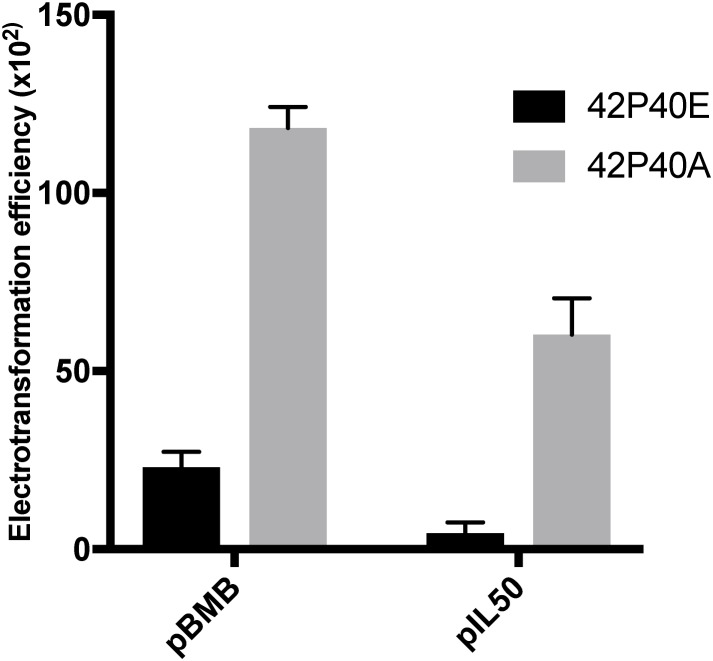
Competence. Electrotransformation efficiency (CFU/μg DNA) of 42P40A (black) and 42P40E (gray) clones after electroporation with plasmids recovered from *S. aureus* (pBMB) or *E. coli* (pLI50) (*p* < 0.001, two-way ANOVA).

A PCR screen detected the *ardA* gene, which is present in the genome of all ST239 comparison genomes, and in the majority (95/100) of isolates from our Brazilian **ST239-BR_100_** collection. The *ardA* gene is also found in other non-ST239 HA-MRSA genomes [e.g., MU50 (ST5-SCC*mec*II)], and in other bacterial pathogens, such as *E. coli* (McMahon et al., 2009). It is important to note that typical CA-MRSA strains, such as USA300 and USA1100, do not harbor this gene, according to BLAST searches in public databases (data not shown). In *E. coli*, the acquisition of *ardA* has a significant impact on gene exchange due to the ability of the ArdA protein to bind and inactivate type I restriction modification (RM) enzymes, which, in turn, results in increased dissemination of MGEs among these bacteria. ArdA from *S. aureus* is active against the *Eco*KI type I RM system of *E. coli* K12 (McMahon et al., 2009), but this has not been demonstrated in *S. aureus*. To test the function of ArdA in *S. aureus*, we cloned the *ardA* gene into the *S. aureus* expression vector pCN40 and transformed it into the *ardA*-negative, ST8, strain RN4220 (42P40A). The ability to acquire exogenous DNA from both *E. coli* and *S. aureus* of a different lineage (ST5) increased significantly (*p* < 0.001) in 42P40A in comparison with 42P40E (RN4220 with empty vector pCN40) (Figure 3).

Recent studies have shown that the high-level multi-resistant ST239 isolates are adapted to the hospital environment (Alp et al., 2009; Xiao et al., 2013). The global dissemination of a bacterial clone is likely to be dependent on specific mechanisms for transmissibility, bacterial adaptability, and fitness (Amaral et al., 2005; Li et al., 2012). Thus, it is reasonable to posit that the presence of the *ardA* gene may represent a substantial evolutionary event in the adaptation to the nosocomial niche, assisting not only in the acquisition of resistance determinants but also of genes associated with virulence/fitness.

#### RGP4

RGP4 is associated with a unique *S. aureus* pathogenicity island (SaPI, Supplementary Figure S2D). SaPIs belong to a large family of phage-inducible chromosomal islands. This SaPI – composed of 14.6 kbp, carries 25 ORFs and is highly similar to SaPITokyo11212. The identified integration site, *att*, belongs to the *att*/*int* IV group (Novick et al., 2010), with the core sequence TTATTTAGCAGGAATAA, which includes the insertion site and an intact ORF for an integrase gene (BMB9393 SABB_00863). Despite the fact that RGP4 and SaPI1 share a homologous integrase, and are located at the same site *att* in the *metQ* gene, they show few similar segments, even in the *rep*/*oriT* sequences (Supplementary Figure S2D). SaPIs can be composed of modular units from other SaPIs (Novick and Subedi, 2007). Indeed, when RGP4 and SaPI2 were compared (Acc: EF010993) there was greater overall similarity than with SaPI1, specifically in the region related to the modules of DNA replication and SaPI packaging.

Interestingly, modular conservation could be related to the fact that SaPI2 is very similar to SaPI1028 (Subedi et al., 2007), for which the “auxiliary” phage is PT1028, which itself is highly similar to the phage found in RGP4. It is important to emphasize that in the ST239-INT_C_ genomes analyzed, the phages related with PT1028 harbor enterotoxin-like genes, such as *selK* and *selQ*, which are absent in all **ST239-BR_C_**. In fact, the **ST239-BR_C_** genomes do not carry any of the known enterotoxin or enterotoxin-like genes, clearly indicating that – despite the role of some enterotoxins in *S. aureus* virulence in animal models (Stach et al., 2014) – the acquisition of these superantigen genes is not crucial for the pathogenesis of severe nosocomial infections including bloodstream infections and other disseminated diseases. On the other hand, the gene encoding the SAP domain-containing protein is lost in all **non-ST239-INTc** strains studied here. This observation is in agreement with the assumption that most SaPIs show an association with specific MRSA lineages instead of widespread distribution among different lineages (Lindsay, 2008).

#### RGP8

This 30.9 kbp region shows similar features to GIs, such as the presence of a putative truncated transposase, *hsd*S (BMB9393 SABB_06193) and *hsd*M (BMB9393 SABB_06194) encoding an RM system, and tRNAs flanking both ends. This GI has been classified as νSAβ type I in ST239 genomes (Baba et al., 2008) and carries the entire serine–protease operon *splABCDEF* (BMB9393 SABB_01932 to SABB_01937) and the gene cluster encoding staphylococcal lantibiotics (BMB9393 SABB_01939 to SABB_05404). In general, *S. aureus* extracellular proteases are considered important virulence factors. For instance, a *S. aureus* mutant derived from USA300 LAC – for which all 10 protease genes were deleted including *spl* proteases – showed lower mortality rates in mice compared with the isogenic wild-type strain (Kolar et al., 2013).

νSAβ is another example of a GI that is widely dispersed in *S. aureus* and not strictly associated with particular lineages, demonstrating that its acquisition may have important adaptive benefits for *S. aureus* strains. Among the strains analyzed, other than JKD6008, only CA347 (USA600) and H-EMRSA-15 do not carry this GI (Supplementary Figure S2E).

#### RGP9

This RGP is 73.2 kbp and corresponds to a bacteriophage-related plasticity region that shows high similarity (BLASTp ≥ 70%) with ϕNM3 (Acc:NC_008617). This region is highly conserved among all ST239-BR_C_ genomes (Supplementary Figure S2F) and is known to be a β-hemolysin converting prophage. RGP9 can also carry the immune evasion cluster (IEC) (Van Wamel et al., 2006), which has also been associated with *S. aureus* host-adaptation and virulence mechanisms (Van Wamel et al., 2006). The previously described *S. aureus* IEC carries genes encoding the chemotaxis-inhibitory protein (CHIPS), staphylococcal complement inhibitor (SCIN), staphylokinase (Sak), and the staphylococcal enterotoxin A (Sea). ST239-BRc genomes have genes for all of these proteins except Sea. We could not detect the *sea* gene in any **ST239-BR_100_** isolates from our collection.

While the *chp* gene is found in all **ST239-BR_C_** strains, it is not found in any of the **ST239-INT_C_** genomes analyzed, raising questions about the specific importance of this gene in the microevolution of Brazilian ST239 (Supplementary Figure S2F). Bae et al. (2006) showed a significant reduction in the ability of *S. aureus* strain Newman to replicate in a murine liver abscess model when ϕNM3, harboring *chp*, was cured. The product of the *chp* gene (BMB9393 SABB_02361), CHIPS, specifically binds to C5a and formylated peptide receptors inhibiting human neutrophils and monocytes chemotaxis (de Haas et al., 2004; Postma et al., 2004). We detected the *chp* gene by PCR in all Brazilian isolates from our **ST239-BR_100_** collection (100%). It is intriguing that in GV69, the IEC cluster including *chp* are carried by a different phage (similar to tp310-3), which may indicate a strong pressure for the acquisition of this gene cluster in Brazilian ST239. Notably, GV69 also does not have the *sea* gene similar to other Brazilian ST239. It is important to note that *chp* is also present in other successful HA-MRSA lineages including ST36 (MRSA252; representative of the EMRSA-16), ST22 (H-EMRSA-15; representative of the EMRSA-16), and ST45 (CA-347; representative of the USA600), within a phage related to ϕNM3. Both *scn* and *sak* are present in all ST239_C_ genomes.

### Virulence Genes

In general, the set of virulence genes in ST239 genomes is well conserved (Supplementary Table S7). The **ST239-BR_C_** and **ST239-INT_C_** genomes harbor a plethora of surface proteins named microbial surface components recognizing adhesive matrix molecules (MSCRAMMs), which are required for the establishment of infections. A notable exception is the absence of genes encoding fibronectin-binding protein B (*fnbB*) and clumping factor A (*clfA*) in T0131 (ST239-INT_C_) and the absence of clumping factor B (*clfB*) in T0131 and XN108 (ST239-INT_C_). These genes are found in all **ST239-BR_C_** genomes (Supplementary Table S7).

Until now, few studies have reported the *tst* gene in ST239 isolates (Iwao et al., 2012; Kong et al., 2016), and most of the enterotoxin and enterotoxin-like genes found in the other HA-MRSA lineages were absent in the ST239 genomes we studied. In fact, the only enterotoxin genes found in any ST239 genomes were *selK*, *selQ*, and *sea*, none of which are found in **ST239-BR_C_**. The reason why it would be an advantage for ST239 isolates to be devoid of enterotoxin genes is unclear, but it is possible that loss of these genes may lead to less cytotoxicity and greater persistence (Das et al., 2016).

The arginine catabolic mobile element (ACME) is a putative virulence determinant, integrated into the chromosome at the same site as SCC*mec* (Diep et al., 2006; Barbier et al., 2011). While there are no reports of ST239 strains carrying ACME I, there are a few reports of ST239 isolates carrying ACME II (Espedido et al., 2012; Hsu et al., 2015). Using a BLAST-based search, we did not find the ACME-encoded *arcA* gene in any of 190 genome sequences analyzed, probably due to the fact that the geographic regions from which ACME+ ST239 strains reported are under-represented in this set of genomes. Nonetheless, ACME does not seem to be important to the biology of ST239 overall.

A unique, conserved cluster consisting of 11 ORFs for putative staphylococcal exotoxin-like genes (*set* or *ssl*) was found in the genomes of **ST239-BR_C_** and **ST239-INT_C_**. Among the other non-ST239-INT_C_ genomes, only USA500 carries a *ssl* gene cluster similar to ST239. Like ST239, USA500 is a member of CC8 and it is possible that this region of *ssl* genes is associated with CC8 evolution (Aguiar-Alves et al., 2006). The role of some *ssl* gene products in impairing neutrophil chemotaxis and complement activation has been reported (Bestebroer et al., 2010; Koymans et al., 2016).

ST239 genomes carry a number of genes conserved in all *S. aureus*, encoding exoproteins likely important for bacterial dissemination in host tissues, such as phospholipases, proteases, hyaluronidase, coagulase, among others. The ST239_C_ genomes are also equipped with several hemolysin-encoded genes including *hla*, *hld*, *hlgABC*, and the leukocidins *lukD* and *lukE*. Interestingly, while JKD6008 has a gene for LukE it lacks the *lukD* gene. As generally observed for canonical hospital strains, the ST239_C_ genomes analyzed do not carry the genes encoding the Panton–Valentine leukocidin (Figueiredo and Ferreira, 2014).

### Mutations in Regulatory Genes

The *agr* operon encodes the main *S. aureus* quorum sensing system (QSS) (Novick et al., 2000), but at least one Brazilian isolate (HC1335) has a transposable element similar to IS*256* disrupting the *agrC* coding sequence (Botelho et al., 2016), an insertion that is expected to inactivate the entire Agr system including multiple virulence determinants, since *agrC* encodes the Agr autoinducer peptide (AIP) receptor. A similar IS*256* insertion was also found in the Chinese ST239 isolate T0131, located 36 nt upstream of the insertion site observed for HC1335 (Supplementary Figure S2G).

Agr dysfunction was assayed in the entire collection of **ST239-BR_100_** isolates using a mass spectrometry approach to detect PSM-*mec*, an Agr-regulated gene. Thirty percent of isolates (*n* = 30) had decreased Agr activity. Most of the Agr-dysfunctional isolates were from colonization cases (*p* < 0.01) (Figure 2B). Among these 30 Agr-dysfunctional isolates, the IS*256* insertion in *agrC* was found by PCR in four isolates (13.3%) suggesting multiple possible inactivation mechanisms. The high prevalence of Agr dysfunction may seem counterintuitive given the prominent role of the Agr system in pathogenicity and virulence (Ferreira et al., 2013), but it is possible that in the specific context of the hospital where patients are debilitated and/or immunocompromised, Agr dysfunction may provide a fitness advantage (Paulander et al., 2012; Baines et al., 2015). However, one important caveat of this analysis is that multiple studies have documented within-patient heterogeneity in Agr dysfunction, and future work should incorporate multiple isolates from the same sample or paired isolates from the same individual.

Point mutations inactivating Agr QSS have predominately been described in the *agrA* or *agrC* genes (Traber and Novick, 2006; Shopsin et al., 2010). To investigate mutational evolution in these genes, we aligned the *agrC* genes of all 190 genomes used for our phylogenetic analysis. This analysis revealed 180 SNPs in the *agrC* genes distributed in 135 genomes, corresponding to 9.0 × 10^-4^ SNPs/nucleotide. The average number of SNPs in several other important global gene regulators (*agrA*, *agrB*, *mgrA*, *rot*, *saeRS*, *sarA*, *sarR*, *sarS*, *sarX*, *sarZ*, and *sigB*) was significantly lower (2.1 × 10^-5^ SNPs/nucleotide) (Supplementary Figure S3). The 180 SNPs in *agrC* genes were located in 10 positions in the *agrC* sequence. Eight SNPs were nonsynonymous mutations. Three nonsynonymous SNPs resulted in amino acid changes, S6R, I311T, and A343T that were conserved in 60, 61, and 40 of the 190 ST239 genomes studied, respectively (Supplementary Figure S3), suggesting that these changes are not strongly detrimental. To test whether substitutions in the *agrC* gene occur at a significantly different frequency compared to the other virulence regulators studied, we used a statistical test based on Poisson probability distribution. The probability that *agrC* mutation rate was the same or lower compared to other genes was extremely low (*p* = 9.4 × 10^-12^) suggesting that this gene may be undergoing more rapid mutational change, a hallmark of possible positive or diversifying selection. No recombination breakpoints were found in *agrC*. Scans for evidence of the action of diversifying selection using two methods (FUBAR and MEME) were in disagreement about selection at codon site 6 (S6R change). FUBAR found evidence for codon site 6 (Prob[d*N* > d*S*] = 0.908, Bayes factor = 12.254), whereas MEME did not (*P* = 0.2). Across the phylogeny, the test for stringency of selection yielded a selection intensity parameter *k* = 1.74 that is usually indicative of an intensification of selection strength (*k* > 1), but it was not significant (*p* = 0.264).

In a recent study that identified two separate clades of ST239 MRSA in Australia, clade 2 also showed *agr*-dysfunction (Baines et al., 2015), which is thought to be due to mutations located upstream of *agr*. In two of the three Agr-dysfunctional **ST239-BRc** genomes, the mutation was not found in the *agr* operon.

Several studies have demonstrated the importance of IS*256* in MRSA evolution. Benson et al. (2014) reported an insertion of IS256 upstream of the repressor of toxins gene, *rot*, in USA500-related strains, leading to a hypervirulent variant of this lineage. Frisch et al. (2018) showed that strains with multiple copies of IS*256* not only grouped together in phylogenetic analysis, but also displayed higher levels of antibiotic resistance. It is notable that in this study the high-level multi-resistant ST239 also showed high copy numbers of IS*256* (12–24 insertions in the ST239-BR_C_ genomes).

It is noteworthy that a copy of an IS*256*-related element was also found inserted at -210 bp of the *mgrA* gene, encoding another global regulatory protein, in the genomes of BMB9393, GV88, Be62, GV51, and HC1335 (ST239-BR_C_) (Supplementary Figure S4). This IS*256* insertion was exclusively found among the Brazilian isolates. It is absent in the genomes of international ST239 strains. *mgrA* is a negative regulator of autolysis and consequently of biofilm development (Ingavale et al., 2003; Gupta et al., 2015). The expression of *mgrA* also has a positive effect on *agr* transcription (Ingavale et al., 2003), which is coordinated by the P1 (more active in stationary phase of growth) and P2 promoters (more active in logarithmic phase) (Ingavale et al., 2003; Gupta et al., 2015). The IS*256* element in the **ST239-BR_C_** genomes was inserted between the P1 and P2 promoters (Supplementary Figure S4). It is notable that *agr*-RNAIII is a key molecule in the stabilization of the 5′-UTR mRNA transcribed under the action of P2*_mgrA_*. Gupta et al. (2015) demonstrated that two adjacent regions transcribed from the upstream P2*_mgrA_*, located between -300 and -200 bp, form a stable complex with two regions of *agr*-RNAIII near its 5′ and 3′-ends. It was suggested that *agr* exerts its regulatory function through MgrA, which could act as an intermediate controller. According to this model, one might expect that the IS*256* insertion near the *mgrA* gene at -210 bp impairs RNAIII-based stability of P2 transcript, thus decreasing the level of *mgrA* transcripts under the coordination of P2*_mgrA_* promoter.

The entire collection of **ST239-BR_100_** isolates was assessed for the IS*256* insertion in the *mgrA* gene using PCR-based detection. Fifty-seven (57%) of the total isolates carry the IS*256* insertion between the *mgrA* promoters. Notably, 43 out of these 57 (75.4%) isolates were from infection-associated cases (*p* < 0.01) (Figure 2C). It has been shown that inactivation of *mgrA* resulted in increased autolytic activity and decreased transcription of several global regulators, including Agr (Luong et al., 2006). Other studies showed that MgrA regulates, directly or indirectly, a total of 355 genes, up-regulating 175 (including genes encoding exoproteins) and down-regulates the remaining 180 genes, among which are genes encoding surface proteins (Ingavale et al., 2005; Luong et al., 2006). Furthermore, the deletion of *mgrA* decreased virulence in an animal model (Jonsson et al., 2008). It is conceivable that the IS*256* insertion found in 57% of ST239-BR isolates would not completely abolish *mgrA* transcription, because this insertion occurred in a position (-210 bp) that would be predicted to affect only P2 transcripts without disturbing P1 regulatory region, located at -28 to +16, which is the binding site for the non-coding RNA, RsaA (Romilly et al., 2014).

### Phylogeny and Divergence Dating of the Three Major ST239 Clades

Maximum-likelihood (Supplementary Tables S2, 10 and Supplementary Figure S5) trees were constructed based on core genome SNPs using 190 complete genomes of ST239 and ST239-like SLVs of both draft and completely closed chromosomes that were available on NCBI during this study-period. Genomes used in the phylogenetic studies by Harris et al. (2010) and Castillo-Ramírez et al. (2012) were also included in this study. Trees were constructed both including and excluding the recombined region thought to be derived from CC30 (corresponding to positions 2848181-3043210 and 1-427978 on the TW20 genome; data not shown), but we used datasets excluding this region for our final analyses. Trees were rooted using outgroups from ST8.

The tree architecture showed three primary ST239 clades referred to here as clades I (*n* = 77 genomes), II (*n* = 33), and III (*n* = 80), that are roughly equivalent to the primary groupings defined in Castillo-Ramírez et al. (2012) (*n* = 165). Both our trees and previously published trees showed a similar branch length distribution with multiple deeply divergent lineages and short branches near the root of the tree possibly suggesting deep and rapid radiation. These deep branches show no consistent geographic location, and they are not restricted to Europe, so it remains unclear where the ST239 lineage originated. This branching pattern also may cause some instability in the placement of early branching taxa in the major early bifurcations. Like previous studies, our tree also showed a similar pattern of three well-defined subclades that had strong geographical affinities and partitioned with long internal branches suggesting local adaptation. For standardization purposes, these subclades were designated as in Harris et al. (2010) and Castillo-Ramírez et al. (2012) as the Asian, South American, and Turkish subclades. All ST239-BR_C_ genomes were found in the main clade II and grouped with genomes mostly from other Latin American and romance language-speaking European countries (South American clade; *n* = 27). ST239-INT_C_ genomes were located in the two other main clades (I and III). Closed genomes of TW20 from the United Kingdom, Z172 from Taiwan, and XN108 from China clustered in clade I, in the Asian subclade (*n* = 63). In clade I, there is a paraphyletic group (*n* = 13) composed largely of strains from Europe (e.g., P32 from Poland, H160 from Hungary, HSA10 from Portugal among others). The ST239-INT_C_ genomes from Australia (JKD6008) and from China (T0131) clustered with the remaining 80 genomes in clade III, in which the derived subclade is represented mostly by Turkish isolates (Turkish subclade; *n* = 70).

**FIGURE 4:**
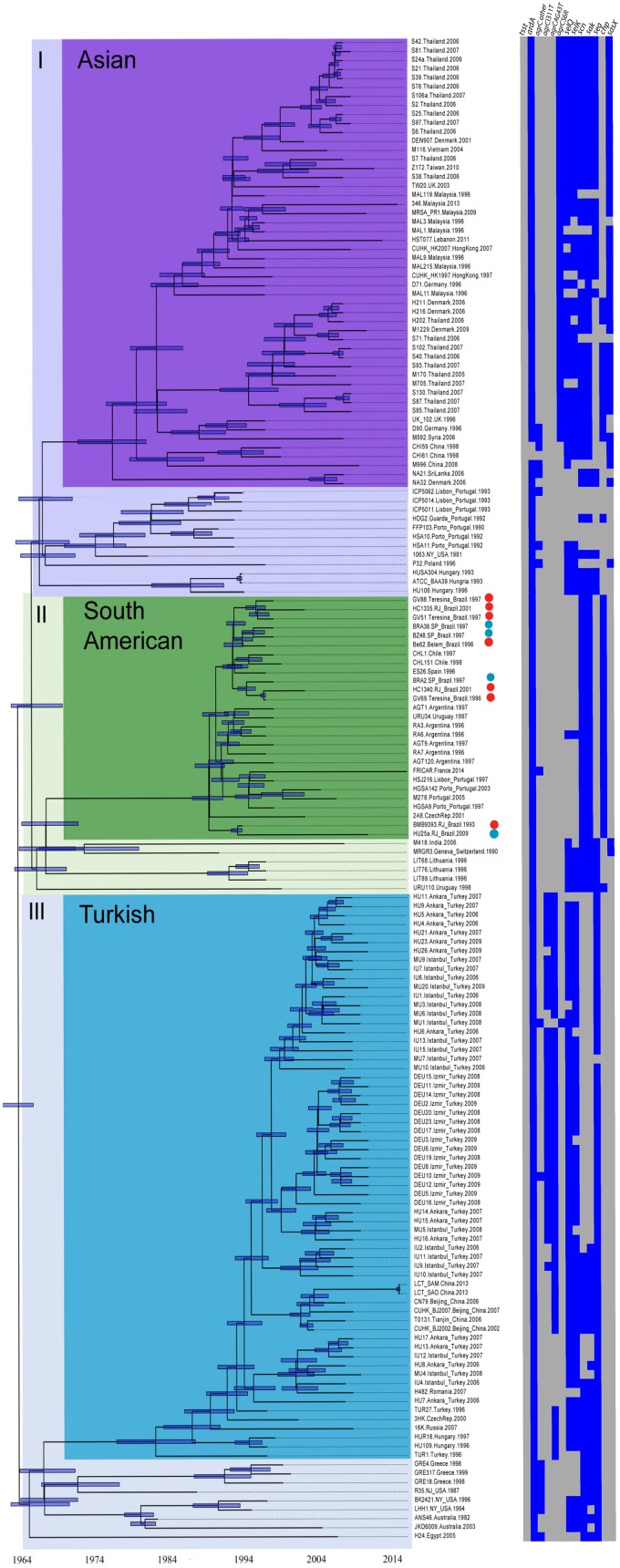
Time-calibrated phylogenetic tree. Maximum clade credibility tree estimated using a log normal-relaxed clock (see the section “Materials and Methods”) from the genomic SNPs revealing three major ST239 clades (I, II, and III), which encompass subclades named as following previous classification: Asian (purple), South American (green), Turkish (light blue). Heat map shows the presence (blue) and absence (gray) of *tsst*, *ardA*, *selQ*, *selK*, *scn*, *sak*, *sea*, *chp*, *sasX*, *chp*, *sea*, and the main SNPs in *agrC* associated with each clade.

For estimation of divergence times using Bayesian estimation (Figure 4), the genomes included were only those for which the isolation date of each isolate was reported (*n* = 167). The resulting Bayesian phylogenetic tree topology, calibrated by incorporating the temporal information of the isolation date and relaxed (Figure 4 and Supplementary Table S8) and strict (Supplementary Figure S7 and Supplementary Table S9) evolutionary clocks, suggests a global initial radiation of ST239 isolates in the 1960s and 1970s. The root of the tree is found at approximately 1962–1966. We estimate that the Asian clade originated in 1976 (HPD 95%: 1972–1980), the South American clade in 1988 (HPD 95%: 1986–1990), and the Turkish clade in 1981 (HPD 95%: 1976–1986). Even more recent intercontinental spread can be inferred based on the branching patterns within each clade. For example, European strains TW20 and D71 from the United Kingdom and Germany, respectively, clustered within the Asian clade; ES26 and M278 from Spain and Portugal, respectively, within the South American clade; and strains T0131 and TN79 from China in the Turkish clade. The intercontinental spread of ST239 isolates has also been documented by Harris et al. (2010) and Gray et al. (2011) among others. Our molecular dating techniques estimate that the expansion of the Brazilian strains began in 1988, coincident with the introduction of the South American clade. Indeed, the first report of ST239 in Brazil was published in 1995 using MRSA isolates collected in the 1992–1994 period (Teixeira et al., 1995). This data are in accordance with previous analyses which estimate that the ST239 introduction into South America occurred in the beginning of the 1990s (Smyth et al., 2010; Castillo-Ramírez et al., 2012). At that time, 87% of the total MRSA isolates detected in multiple Brazilian hospitals from the South to the North of the country belonged to the ST239 lineage, and all of them displayed the same or very similar PFGE patterns, suggesting a very recent clonal spread (Teixeira et al., 1995).

### Virulence Profile of Different Phylogenetic Groups

Local BLAST was performed to compare all 190 ST239 genomes in relation to virulence-associated genes that diverged or were not homogeneously present in the virulome of the ST239_C_ genomes. Our analyses revealed that several genes are associated with specific clades on the phylogenetic trees. The *chp* gene is present in 28 of 33 (81.8%) genomes of clade II (where the South American subclade is located). There is only one genome (URU110; Uruguay-1998; clade II) among the 190 genomes that carries both *chp* and *sea* genes, and all others carrying *chp* lack *sea* (81.8%, *n* = 27). Only five genomes in clade II lack *chp* and carry *sea* (M418, India-2006; MRGR3 from Switzerland-1990; LIT68, LIT89, and LIT76 from Lithuania-1996). These genomes together with URU110 are found in a paraphyletic group in a basal position of clade II. Among all 190 genomes analyzed, only 17 do not carry *chp* or *sea* (Supplementary Table S2).

Clade I is composed of a total of 77 genomes, and the great majority, 75.6% (*n* = 59), carry the *sasX* gene encoding a LPXTG motif protein. All of these group in the Asian subclade, except for one genome (P32 from Poland) located in a basal position in this clade. SasX promotes bacterial aggregation, enhances nasal colonization and immune evasion, and plays a role in lung infection and abscess formation in animal models (Li et al., 2012). Outside the clade I, only two other ST239 genomes carry *sasX*, M418 (India, 2006), and MRGR3 (Switzerland, 1990). Both of these group at the base of clade II. Thus, it is possible that the common ancestor (dated to approximately 1966, 95% HPD 1963–1970) of the Asian and South American subclades carried *sasX* but, after local diversification, only the Asian clade conserved this gene.

There are only four genomes (5.1%) in the Asian subclade (M592, Syria-2006; D90, Germany-1996; United Kingdom 102, UK-1996; and M170, Thailand-2005) where the *chp* gene shows 100% identity with the *chp* sequence found in the South American subclade (*chp-1* allele). In addition, in clade I, there are an additional 11 genomes (S40, S85, S87, S93, S102, S130, H202, M705, from Thailand-2007; H211, H216, from Denmark-2006; HDG2, from Portugal-1992) in which the *chp* gene displays up to four non-synonymous SNPs compared to the reference (variant *chp-2*). The genomes with *chp-2* are all found in the Asian subclade except for the Portuguese strain HDG2 that is located in a basal position in this subclade (Supplementary Table S2). Most Asian-subclade *chp*-2 positive strains also carry *sasX* genes (12/15), highlighting the acquisition of *sasX* as an important divergent evolutionary event for the Asian subclade. Many of the genomes grouped in clade I also carry *sea* (60.3%, *n* = 47). The genome of the strain OC3 (Russia, 2007–2009) is the only genome in this clade to harbor the *tst* gene (Supplementary Table S2). For all 80 genomes in clade III (where the Turkish subclade is located), both *sasX* and *chp* are absent, and *sea* is present in all but one genome (MU7) from Turkey-2007 (97.5%, *n* = 78) (Figure 4).

While *agr*-dysfunction was detected in 30% of the ST239 isolates from the Brazilian collection, the large majority of the genomes found in clade II show no variants in *agrC* (*n* = 32, 97%). Only one genome (RA3, Argentina-1996) shows any variation, a substitution of glutamic acid (E) for lysine (K) at position 175. None of the genomes found in the two other main clades share this point mutation.

Variants in *agrC* in the other two main clades (I and III) are also well conserved. The nonsynonymous variant A343T (*n* = 58) and I311T (*n* = 61) in the AgrC protein are found in 74.4 and 78.2% of the genomes grouped in the clade III. Another AgrC nonsynonymous variant S6R (*n* = 39) is specifically detected among many strains from clade I (48.1%), although one strain (MU1) from Turkey (clade III) also has this substitution. The *agrC* variants reported here are distinct from positions reported in other studies involving non-ST239 MRSA lineages (Geisinger et al., 2009; Shopsin et al., 2010).

Grouping genomes by their virulence gene patterns, based on absence or presence of *sea*, *chp*, and *sasX* genes and *agrC* SNPs (Figure 4), we used the Grubbs’ test statistic to detect outliers and find a predominant virulence profile for each phylogenetic group (Supplementary Figure S6). Accordingly, the predominant pattern of each group was clade I: *sasX^+^*, *chp^-^*, *sea*^+^, *agrC* S6R^+^, *agrC* I311T*^-^*, *agrC* A343T*^-^*; clade II: *sasX^-^*, *chp*^+^, *sea^-^*, *agrC* S6R*^-^*, *agrC* I311T*^-^*, *agrC* A343T*^-^*; and clade III: *sasX^-^*, *chp^-^*, *sea*^+^, *agrC* S6R*^-^*, *agrC* I311T^+^, *agrC* A343T^+^; Each of these patterns and corresponding SLVs were strongly associated (χ^2^-square, *p* < 0.0001; Supplementary Figure S6) with each of the three main clades and, independently, with broad geographical location of isolation. Gene patterns from a geographic origin were significantly independent from those from another region (*p* < 0.0001). This pattern strongly suggests the geographic (allopatric) influence on the diversification of ST239 MRSA. The selective pressures that may have driven this diversification remain unknown. It is possible that pathogen–immune system and pathogen–microbiome interactions play a role, in addition to broad pressures related to medical practices and socio-cultural influences (Georgiades and Raoult, 2011).

## Conclusion

Our genomic and phylogenetic analysis of the ST239 lineage revealed specific patterns of local geographic divergence involving well-known virulence factors. The genomes grouped with the Brazilian ST239 isolates show a specific virulence pattern represented by the presence of *chp-1*, lack of *sasX* and *sea* (both of which are commonly observed in genomes grouped in the Asian subclade). In addition, we also found that the *ardA* gene was present and conserved in all 190 ST239 genomes analyzed, and confirmed ArdA-antirestriction activity in *S. aureus*, resulting in high-frequency plasmid-borne acquisition of resistance genes. We posit that the *ardA* gene played a role in the acquisition of high-level antimicrobial resistance and the plethora of virulence genes observed in this successful HA-MRSA lineage. Further we posit that the presence of this gene might have facilitated the pattern of local diversification seen in each of the major ST239 subclades, allowing each to rapidly adapt to its local context.

Mobile genetic elements such as phages and GIs have clearly played an important role in the evolution of virulence of this MRSA lineage. The insertion of transposable elements (derived from IS*256*) in, or close to, the important global regulatory genes such as *agr* and *mgrA*, may also function as an additional strategy for MRSA evolution, representing a rapid mechanism for global reprogramming of virulence attributes (Benson et al., 2014). Mutations driving *agr* dysfunction, represented by point mutation or IS*256* insertion in the *agrC* gene, and also mutations upstream of the *agr* locus may be instances of convergent functional evolution, that may have decreased toxicity, and increased colonization and persistence (Le and Otto, 2015; Edmiston et al., 2016), balancing the increased fitness costs of antimicrobial resistance or other nosocomial adaptations.

Finally, much of the diversification we identified in the three major ST239 subclades appears to have occurred in the 1970–1980s, when MRSA emerged causing hospital outbreaks in Europe, United States, and Australia (Aires de Sousa et al., 1998). To more fully understand the process of virulence gene diversification it is possible that targeted efforts on banked culture or tissue samples may more fully reveal the process of bacterial adaptation. Further studies might also be focused on uncovering the selective processes that favored one set of virulence-associated genes over another in each of the three major areas of diversification, and also in understanding evolution in other geographical locations or countries that have been so far neglected. It is clear that broad taxon sampling is critical to unraveling diversity patterns and processes that drive global radiations, allopatric diversification, or multigeographic shifts in pathogens.

## Author Contributions

AB carried out most of the experiments in this study, manual annotation, in addition to bioinformatics analyses using Local BLAST and Geneious. AMNB wrote the draft manuscript. MC and MN manual annotation, genome architecture, and RGP analysis. FF, MC, BC, and PB manual annotation. DS and CB manual annotation, *ardA* molecular cloning and transformation experiments with *ardA*-positive and -negative isogenic clones. RS, LA, and AV sequencing experiments, genome assembly, run automatic annotation, and other bioinformatics analyses. AN, AM, PP, and KO’B phylogenetic analysis. S-OK, AM, and PP phylogenetic analysis, divergence times estimation, recombination scan and analysis, and natural selection scan. AF responsible for the study design. PP and AF wrote the final version of the manuscript. All others contributed to and reviewed the final version of the manuscript.

## Conflict of Interest Statement

The authors declare that the research was conducted in the absence of any commercial or financial relationships that could be construed as a potential conflict of interest.

## Footnotes

1 https://www.ncbi.nlm.nih.gov/genome/genomes/154?genome_assembly_id=biotreegroup16223, last accessed in 18 April 2018.

2 http://mummer.sourceforge.net

3 https://github.com/lh3/wgsim

4 https://github.com/tseemann/snippy

5 https://github.com/thanhleviet/maskrc-svg

6 http://beast.bio.ed.ac.uk/Tracer

7 http://tree.bio.ed.ac.uk/software/figtree/

8 http://datamonkey.org

## Supplementary Material

Figure SI An illustration of the assembly and gap-closing strategies. Gaps intra- and inter-scaffolds were resolved using an hybrid assembly strategy with the Newbler and GapFiller programs, with local assemblies in gap regions using Newbler.

Figure S2 Regions of Genetic Plasticity. Graphical representation of linear comparative alignments. Arrows represent predicted genes and their respective transcription strands (forward/reverse). Vertical red segments connecting regions represent direct conserved segments (BLASTN > 70%). Plots above each region indicate the corresponding % G+C content. Figures were generated using Easyfig program. **(A)** Chromosome region harboring the *atl gene* in BMB9393 (ST239 closed genome; Brazil) and TW20 (ST239 closed genome from United Kingdom). *atl* is highlighted in green. **(B)** SCCmec type III for BMB9393 (ST239 closed genome; Brazil) and TW20 (ST239 closed genome; United Kingdom) sequences. Genes coding for the recombinases are represented in blue; *mecA* and *mecRI*, in dark and light pink, respectively; and IS437 elements, in green. **(C)** RGP3 context for MU50 (non-ST239 closed genome, Japan), BMB9393 (ST239 closed genome; Brazil) and TW20 (ST239 closed genome; United Kingdom) sequences. The *ardA* gene, which encodes for an antirestriction protein, is represented in yellow. Strain BMB9393 lacks genes for an integrase and a hypothetical protein. **(D)** RGP4 carrying a unique SaPI. **(top)** BMB9393 (ST239 closed genome; Brazil) and SaPM sequences, **(bottom)** BMB9393 and SaPI2 sequences. In light pink, gene encoding an integrase; in light green, gene encoding an excisionase; in brown, *pri* genes; in dark green, *rep* gene; in dark blue, p/fgene; in light blue, operon required to DNA packaging; in dark pink, gene encoding a recombinase protein. **(E)** RGP8 carrying the vSAp genomic island using the following genomic sequences: JKD6008 (ST239 closed genome; Australia), BMB9393 (ST239 closed genome; Brazil) and MRSA252 (non-ST239 closed genome; United Kingdom). In green, *hsdS* and *hsdM*; in pink, the gene cluster encoding for serine proteases; in yellow, the gene cluster encoding for staphylococcal lantibiotics. **(F)** RGP9 carrying a bacteriophage related to PhiNM3 in the genomes of BMB9393 (ST239 close genome from Brazil; ST239-BR_C_) and TW20 (ST239 closed genome from United Kingdom; ST239-INT_C_). Arrows represent predicted ORFs and their respective transcription strands (forward/reverse). In light blue, gene encoding a prophage integrase; in blue, *hlb* encoding a trucated p-hemolysin; in yellow, *chp* gene encoding a chemotaxis inhibitory protein (exclusively found in ST239-BR_C_); in green and pink, *sen* and *sak* genes encoding the staphylococccal complement inhibitory protein SCIN and staphylokinase; and in purple *sea* encoding staphylococcal enterotoxin A (absent in BMB9393 and present in TW20). **(G)** *agr* operon for the genome of the strains BMB9393 (ST239 closed genome from Brazil; ST239-BR_C_), HC1335 (ST239-BR_C_) and T0131 (ST239 closed genome from China; ST239-INT_C_). Arrows in blue *hid* (delta-toxin); *agrB] agrD; agrC* (blue); *agrA* and the transposase (yellow).

Figure S3 Substitutions in agrC vs. other global gene regulatiors. **(A)** SNP allele frequency found in pools of DNA from the genomes of 190 ST239 deposited om the Genbank for the regulators of agrC (blue circle), agrA, rngrA, rot, sacRS, sarA, sarR, sarS, sarXy sarZ, and sigB (other colored cirlces.) The horizontal line represents the average frequency. The probability *P* = 9.4x×lOA-12 is the chance of a SNP in agrC to occur at the same mean frequency observed for the other regulators using Poisson distribution. **(B)** Graphical representation shows the amino acid change and its position in the AgrC protein. In parenthesis is the number of genomes that show the correspondent SNP. From the 10 types of SNP substitution found, eight resulted in nonsynonymous changes.

Figure S4 IS256 insertion at mgrA locus. Schematic representation of the rngrA gene regulator showing the position of the insertion of the IS256 between P2 (log-phase promoter) and PI (stationary-phase promoter).

Figure S5 Maximum likelihood tree. Patterns within the three clades are denoted by a darker shade of the parent clade color: Asian pattern as purple, South American pattern as green, and Turkish pattern as blue. See methods for tree construction parameters. Red circles denote an isolate from this study while blue circles denote an isolate obtained from GenBank.

Figure S6 Virulence gene patterns in clades. Distribution of the predominant pattern and its one-locus variants (clade specific patterns) among the genomes clustered in the Type I, Type II and Type III clades. Turkish predominant pattern (*sosX*^1^, *chp*^-^, *sea*^+^, *agrC* S6R^-^, *agrC* I311T^+^ *agrC* A343T^+^), South American predominant pattern (*sasX*, *chp*^+^, *sea*^1^, *agrC* S6R^∼^, *agrC* I311T, *ogrC* A343T), Asian predominant pattern *sasX*^+^, *chp*^-^, *sea*^+^, *agrC* S6R^+^, *agrC* I311T, *agrC* A343T). Other patterns comprises all patterns that differed by two or more loci from the specific clade pattern. The distribution of the clade specific patterns was extremely significant associated with the tree topology and geographic location of the strains. ^∗∗∗^ = (*p* < 0.0001).

Figure S7 A Bayesian phylogenetic tree based on core genome SNPs yields three distinct ST239 patterns (named based on predominant locations of strains): Turkish (light blue), South American (green) and Asian (purple). The Bayesian tree was constructed using r 190 and 167 complete genomes of ST239 and ST239-like single locus variants (SLVs) of both draft and completely closed chromosomes (obtained from NCBI) as well as genomes used in the phylogenetic studies by Harris et al). A heat map shows the presence (grey)/absence (blue) of *sasX*, *chp*, *sea* and main SNP mutation in *agrC* associated with each clade.

Table S1 Main characteristics of the ST239-BR100 strains.

Table S2 List of genomes used on ML analysis and their main characteristics.

Table S3 Primers used on PCR assays in this work.

Table S4 Sequences that were potentially mobile excluded from SNP dataset.

Table S5 Exclusive clusters comparing ST239-BRC and ST239-INTC.

Table S6 Description of RGPs main features.

Table S7 Virulence set of ST239-BRC, ST239-INTC and non-ST239-INTC.

Table S8 BEAST uncorrelated, lognormal tree.

Table S9 BEAST strict clock tree.

Table S10 Maximum likelihood tree.

## Abbreviations

CA-MRSA: community-acquired methicillin-resistant *Staphylococcus aureus*
CC: clonal complex
HA-MRSA: hospital-acquired methicillin-resistant *Staphylococcus aureus*
IS: insertion sequence
MALDI–TOF–MS: matrix-assisted laser desorption/ionization time-of-flight mass spectrometry
MGE: mobile genetic element
MRSA: methicillin-resistant *Staphylococcus aureus*
RGP: region of genomic plasticity
SCC*mec*: staphylococcal cassette chromosome *mec*
SET: staphylococcal enterotoxin-like proteins
SNP: single-nucleotide polymorphism
SSL: staphylococcal superantigen-like proteins
ST: sequence typing
TE: transposase element
