# Supplementary figures and images for: Local Diversification of Methicillin- Resistant Staphylococcus aureus ST239 in South America After Its Rapid Worldwide Dissemination
Source: Front Microbiol. 2019 Feb 27;10:82. doi: 10.3389/fmicb.2019.00082 (PMC6400870; doi:10.3389/fmicb.2019.00082)

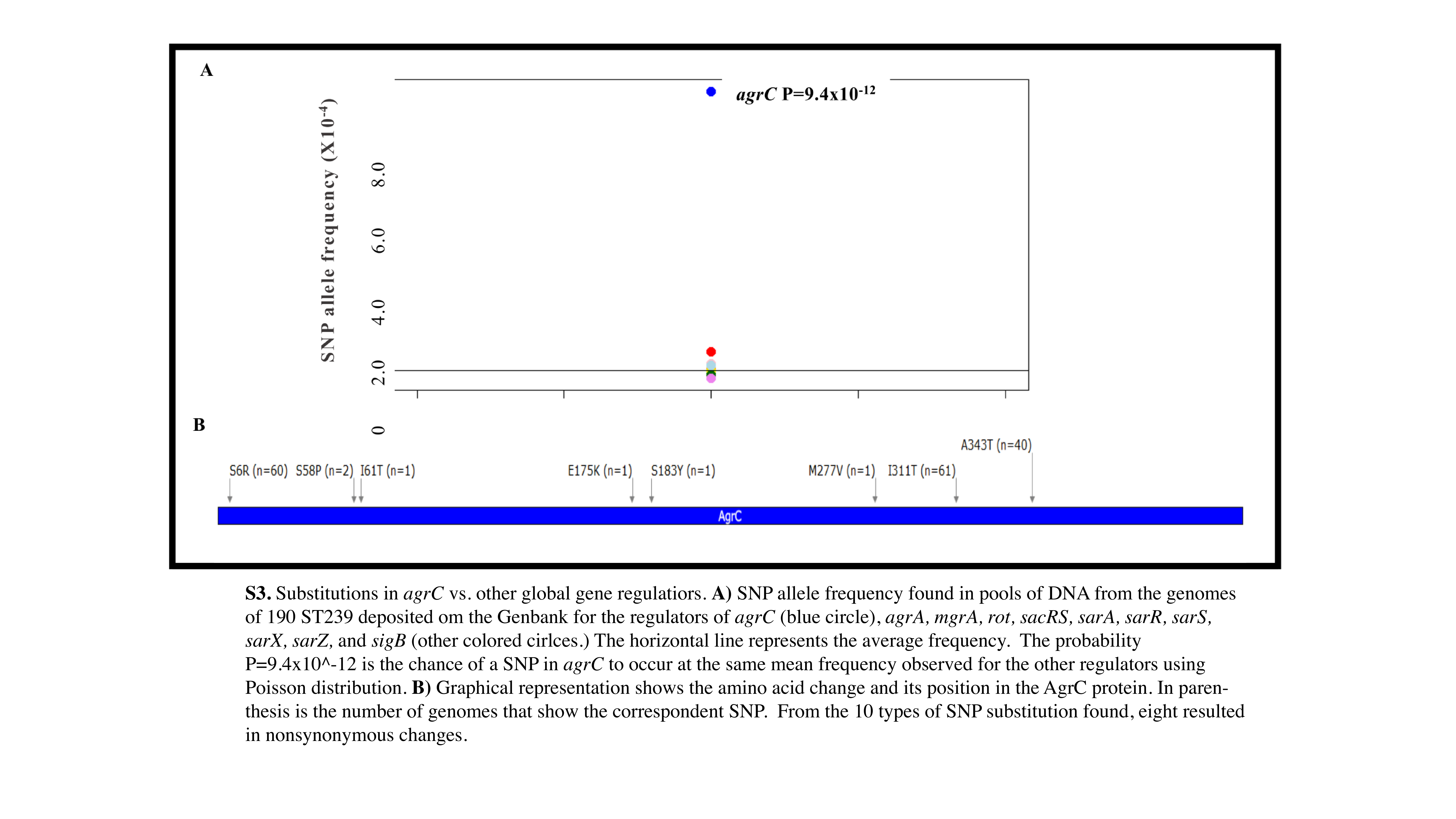

Supplement: Figure S3 — Substitutions in agrC vs. other global gene regulatiors. (A) SNP allele frequency found in pools of DNA from the genomes of 190 ST239 deposited om the Genbank for the regulators of agrC (blue circle), agrA, rngrA, rot, sacRS, sarA, sarR, sarS, sarXy sarZ, and sigB (other colored cirlces.) The horizontal line represents the average frequency. The probability P = 9.4x×lOA-12 is the chance of a SNP in agrC to occur at the same mean frequency observed for the other regulators using Poisson distribution. (B) Graphical representation shows the amino acid change and its position in the AgrC protein. In parenthesis is the number of genomes that show the correspondent SNP. From the 10 types of SNP substitution found, eight resulted in nonsynonymous changes. [file Image_3.TIFF]

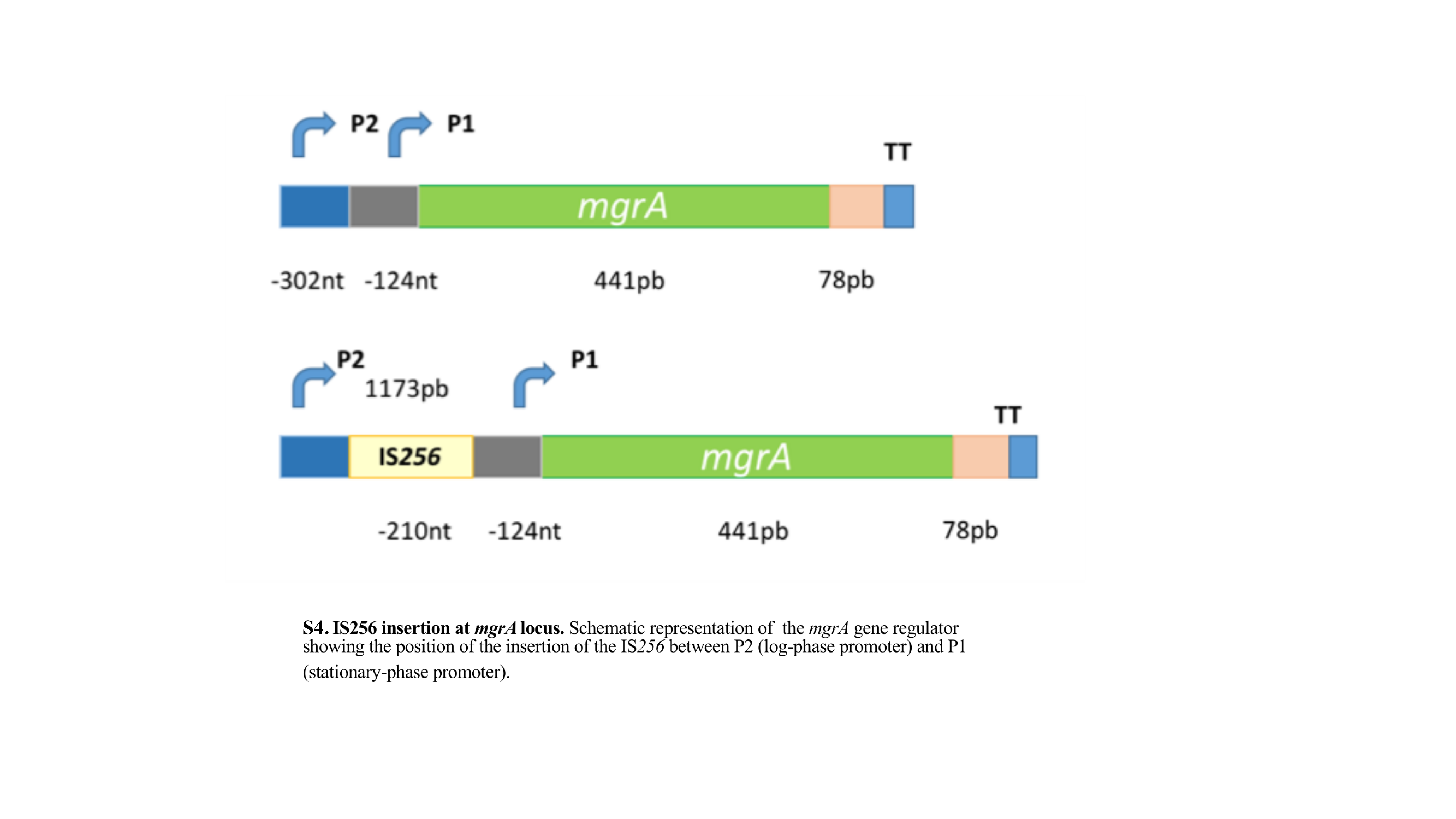

Supplement: Figure S4 — IS256 insertion at mgrA locus. Schematic representation of the rngrA gene regulator showing the position of the insertion of the IS256 between P2 (log-phase promoter) and PI (stationary-phase promoter). [file Image_4.TIFF]

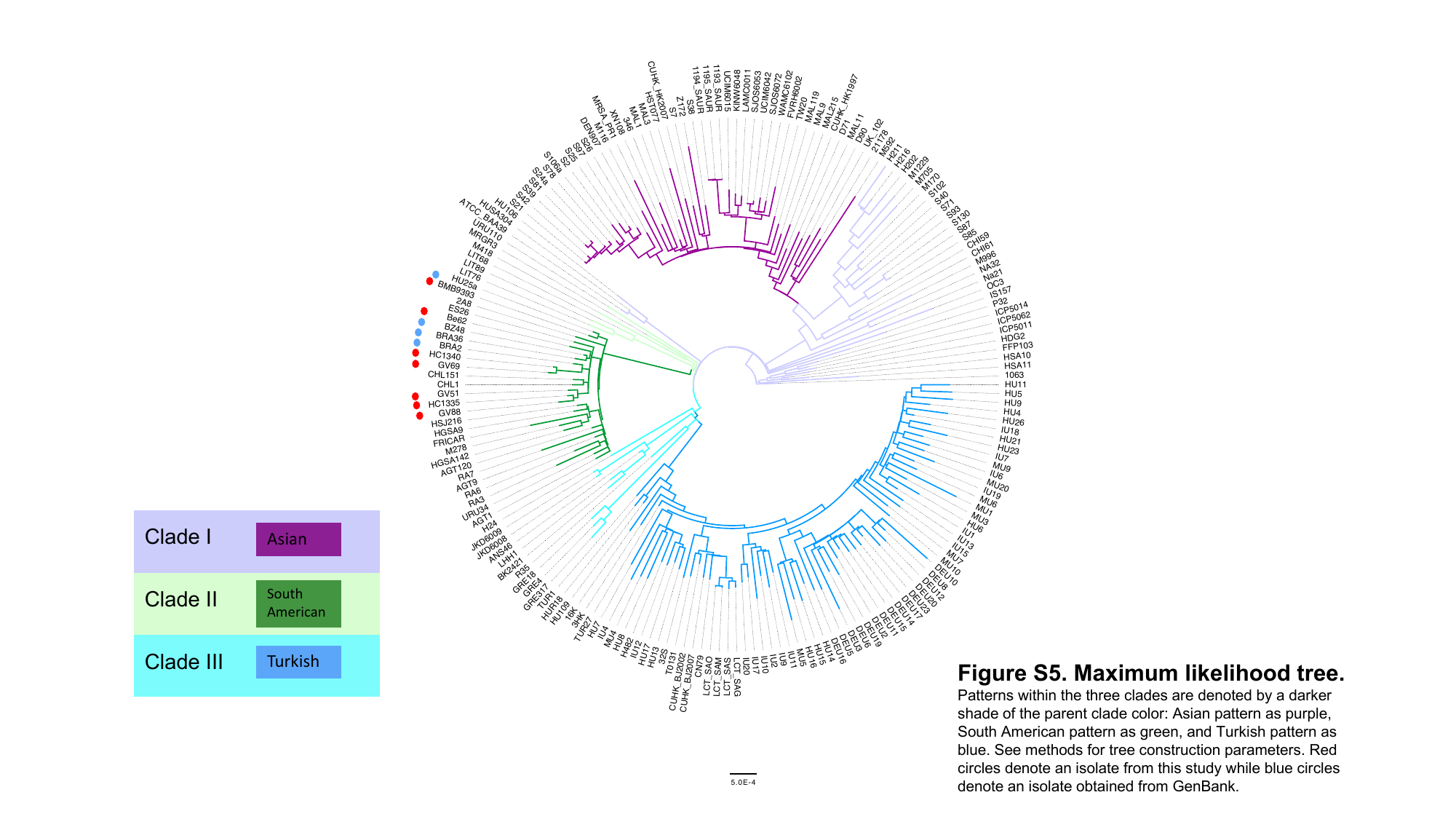

Supplement: Figure S5 — Maximum likelihood tree. Patterns within the three clades are denoted by a darker shade of the parent clade color: Asian pattern as purple, South American pattern as green, and Turkish pattern as blue. See methods for tree construction parameters. Red circles denote an isolate from this study while blue circles denote an isolate obtained from GenBank. [file Image_5.TIFF]

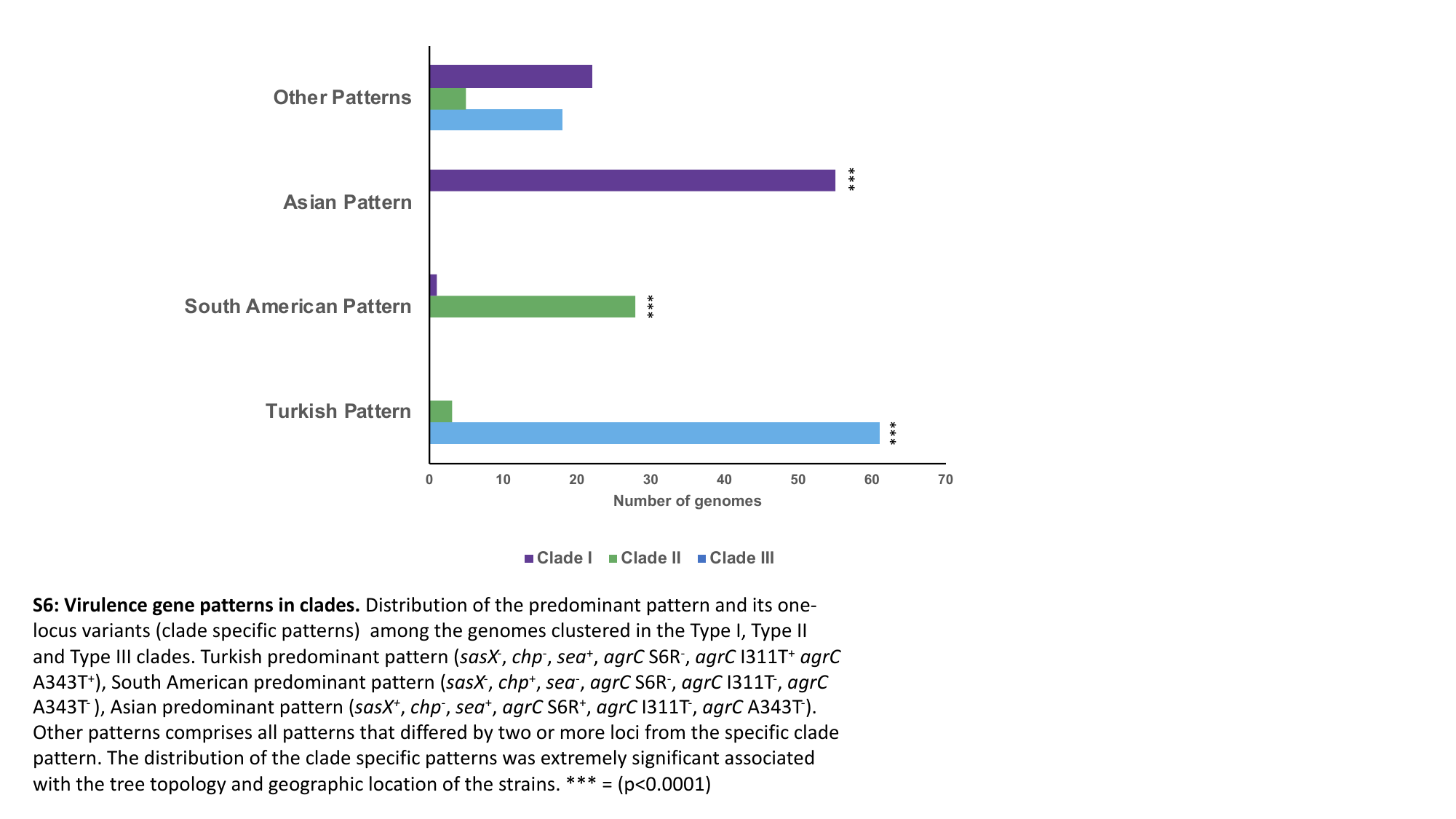

Supplement: Figure S6 — Virulence gene patterns in clades. Distribution of the predominant pattern and its one-locus variants (clade specific patterns) among the genomes clustered in the Type I, Type II and Type III clades. Turkish predominant pattern (sosX1, chp-, sea+, agrC S6R-, agrC I311T+ agrC A343T+), South American predominant pattern (sasX, chp+, sea1, agrC S6R∼, agrC I311T, ogrC A343T), Asian predominant pattern sasX+, chp-, sea+, agrC S6R+, agrC I311T, agrC A343T). Other patterns comprises all patterns that differed by two or more loci from the specific clade pattern. The distribution of the clade specific patterns was extremely significant associated with the tree topology and geographic location of the strains. ∗∗∗ = (p < 0.0001). [file Image_6.TIFF]

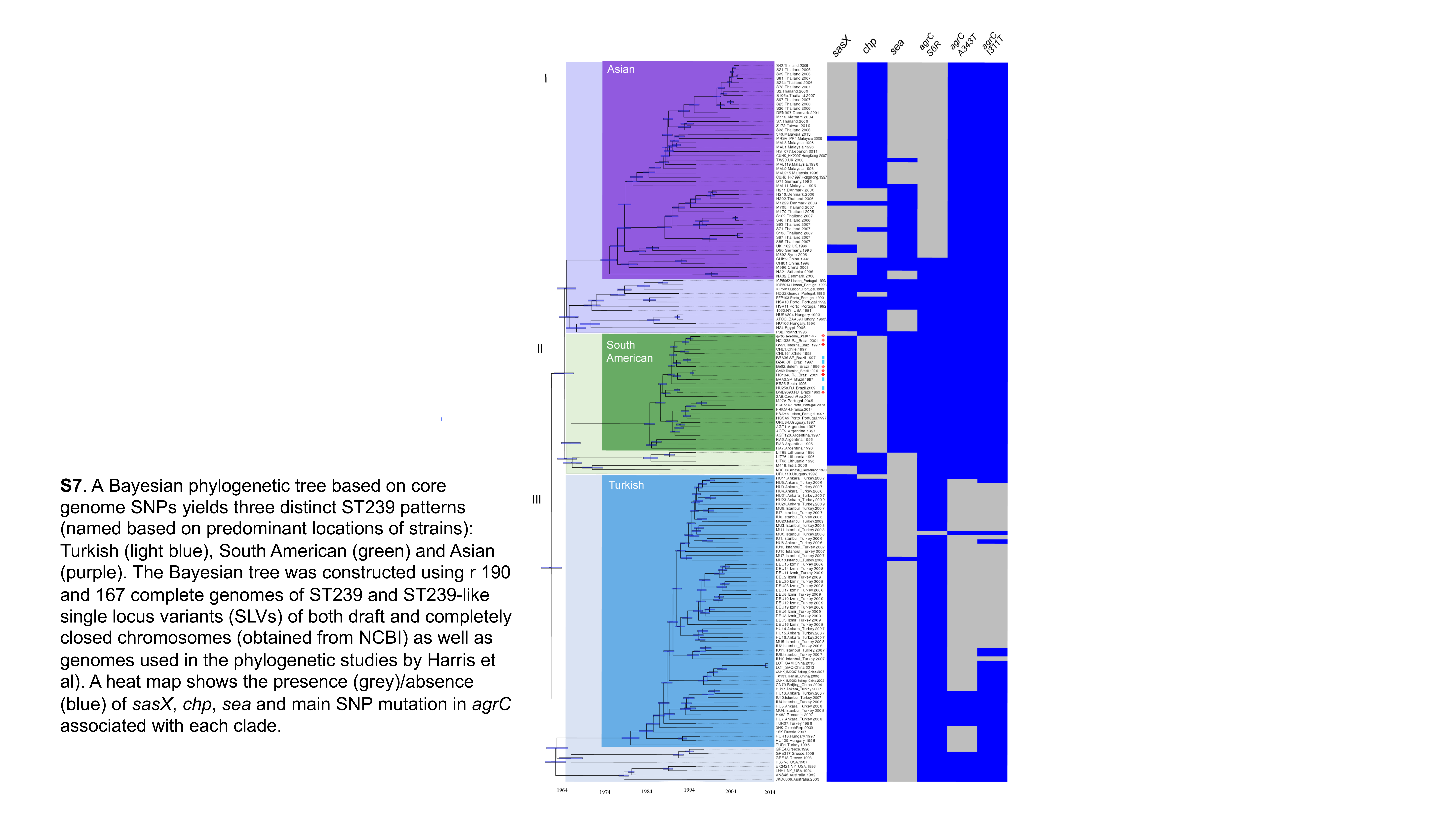

Supplement: Figure S7 — A Bayesian phylogenetic tree based on core genome SNPs yields three distinct ST239 patterns (named based on predominant locations of strains): Turkish (light blue), South American (green) and Asian (purple). The Bayesian tree was constructed using r 190 and 167 complete genomes of ST239 and ST239-like single locus variants (SLVs) of both draft and completely closed chromosomes (obtained from NCBI) as well as genomes used in the phylogenetic studies by Harris et al). A heat map shows the presence (grey)/absence (blue) of sasX, chp, sea and main SNP mutation in agrC associated with each clade. [file Image_7.TIF]
