# Supplementary material for: Local Diversification of Methicillin- Resistant Staphylococcus aureus ST239 in South America After Its Rapid Worldwide Dissemination
Source: Front Microbiol. 2019 Feb 27;10:82. doi: 10.3389/fmicb.2019.00082 (PMC6400870; doi:10.3389/fmicb.2019.00082)
Supplement: Table S6 — Description of RGPs main features. [file Table_6.DOC]

**- Regions of genomic plasticity**

**RGP2.** The most relevant feature of this region is the presence of the gene cluster *aad*E–*sat*4–*aph*A-3, which encodes two aminoglycosides-modifying enzymes (AMEs). RGP2 has variable organization among all MRSA genomes analyzed, but it is significantly conserved in the ST239-BR_C_.

**RGP5.** The RGP5 is a bacteriophage-related plasticity region of 48.5 Kbp, which is more conserved in ST239-BR_C_ genomes. Nevertheless, it shows more syntenic regions with the genomes of the MRSA252 and CA-347 strains than with the other compared genomes, including the ST239-INT_C_.

**RGP6. This region** corresponds to 13.5 Kbp flanked in the initial portion by an IS*256* (BMB9393 SABB_01339). This region contains the *cls1* gene encoding the cardiolipin synthase 1, responsible for the accumulation of intracellular cardiolipin. This region is highly conserved among all compared genomes except for the isolate HC1335 (ST239-BR), which lost the *cls1* gene.

**RGP7.** This is another bacteriophage-related plasticity region with 51.8 Kbp of size (Table 3). This region shows more similarity with the *Staphylococcus* phage ϕMR11 (GenBank Accession Number: NC_010147) than with other 23 phage species identified by PHAST and BLASTp tools.

**RGP8.**  This region of 30.9 Kbp shows similar features to GIs, such as the presence of a truncated transposase, *hsd*S (BMB9393 SABB_01927) and *hsd*M (BMB9393 SABB_01928) encoding for a RM system, and tRNAs flanking both ends. This GI in the ST239 genomes was classified as νSAβ type I, carrying the whole serine-protease operon *splABCDEF* (BMB9393 SABB_01929 to SABB_01934) and the gene cluster encoding the staphylococcal lantibiotics (BMB9393 SABB_01936 to SABB_01944). For this region either a synteny conservation among strains belonging to different clones (all ST239 and the USA500 strain 2395) or a synteny break intra-linage [JKD6008 (ST239-INT) and all ST239] since JKD6008 do not carry this GI (Figure S1). The extracellular proteases is considered an important virulence factor. A *S. aureus* mutant derived from USA300 LAC—for which all 10 proteases genes present in the genome of the *S. aureus* were deleted— showed lower mortality rates in mice compared with wild-type strain. Among the isolates analyzed, besides the JKD6008, only the isolates CA347 (USA600 clone) and H-EMRSA-15 (EMRSA-15 clone) do not carry this GI. The νSAβ is an example of a GI that is not strictly associated to particular linages.

**RGP10.** This region contains 5.4 Kbp and corresponds to a plasticity region associated to transposable elements, which carries *aac*(6′)/*aph*(2″) (BMB9393 SABB_02184), another gene encoding a cellular aminoglycosides-modifying enzyme (AME), which mediates resistant to aminoglycosides in staphylococci. The gene for this bifunctional AMEs is part of a composite transposon Tn*4001*, which is flanked by two identical insertion sequences IS*256* (BMB9393 SABB_02183 and SABB_02186). Tn*4001* is absent in the non-ST239_C_ genomes of Mu50, CA-347 (Figure 3a), MRSA252 and H-EMRSA-15 strains.

**RGP 11.** This RGP of 25.8 Kbp is also related to a transposable element and flanked by an incomplete resolvase (BMB9393 SABB_02272) and a IS*1181* family transposase belonging to in the initial region and by tRNAs in the final region. (BMB9393 SABB_02286) Among other genes, RGP11 harbors a *fmtB* gene (BMB9393 SABB_02281) encoding for a cellular surface protein FmtB containing a multiple repeats domain and a LPXTG motif-containing region at the C-terminal region, whose inactivation reduced methicillin resistance.

**RGP 12.** This region has 20.2 Kbp and harbors an integrative and conjugative element similar to ICE*6013*, which was previously identified in other *S. aureus* belonging to the ST239 lineage. The ICE*6013*-related element is fairly conserved among all compared ST239_C_ genomes. In contrast, this element is missing in the non-ST239_C_ genomes of the strains Mu50 and CA-347.
